# Myeloid cell-derived inducible nitric oxide synthase suppresses M1 macrophage polarization

**DOI:** 10.1038/ncomms7676

**Published:** 2015-03-27

**Authors:** Geming Lu, Ruihua Zhang, Shuo Geng, Liang Peng, Padmini Jayaraman, Chun Chen, Feifong Xu, Jianjun Yang, Qin Li, Hao Zheng, Kimberly Shen, Juan Wang, Xiyu Liu, Weidong Wang, Zihan Zheng, Chen-Feng Qi, Chuanping Si, John Cijiang He, Kebin Liu, Sergio A. Lira, Andrew G. Sikora, Liwu Li, Huabao Xiong

**Affiliations:** 1 Department of Medicine, Immunology Institute, Icahn School of Medicine at Mount Sinai, New York, New York 10029, USA; 2 Department of Biological Sciences, Center for Inflammation, Virginia Tech, Blacksburg, Virginia 24061, USA; 3 The Ronald O. Perelman and Claudia Cohen Center for Reproductive Medicine, Weill Medical College of Cornell University, New York, New York 10021, USA; 4 Laboratory of Immunogenetics, National Institute of Allergy and Infectious Diseases, National Institutes of Health, Bethesda, Maryland 20892, USA; 5 Institute of Immunology and Molecular Medicine, Jining Medical College, Jining, Shandong 272067, China; 6 Department of Biochemistry and Molecular Biology, Medical College of Georgia, Georgia Regents University, Augusta, Georgia 30912, USA

## Abstract

Here we show that iNOS-deficient mice display enhanced classically activated M1 macrophage polarization without major effects on alternatively activated M2 macrophages. eNOS and nNOS mutant mice show comparable M1 macrophage polarization compared with wild-type control mice. Addition of N6-(1-iminoethyl)-L-lysine dihydrochloride, an iNOS inhibitor, significantly enhances M1 macrophage polarization while S-nitroso-N-acetylpenicillamine, a NO donor, suppresses M1 macrophage polarization. NO derived from iNOS mediates nitration of tyrosine residues in IRF5 protein, leading to the suppression of IRF5-targeted M1 macrophage signature gene activation. Computational analyses corroborate a circuit that fine-tunes the expression of IL-12 by iNOS in macrophages, potentially enabling versatile responses based on changing microenvironments. Finally, studies of an experimental model of endotoxin shock show that iNOS deficiency results in more severe inflammation with an enhanced M1 macrophage activation phenotype. These results suggest that NO derived from iNOS in activated macrophages suppresses M1 macrophage polarization.

## Abstract (web summary)

In response to microbial ligands, IRF5 promotes pro-inflammatory M1 macrophage activation and production of nitrous oxide. Here the authors show that nitrous oxide modifies IRF5 tyrosine residues as a negative feedback, limiting the inflammatory response and protecting from endotoxin shock.

## Graphical abstract

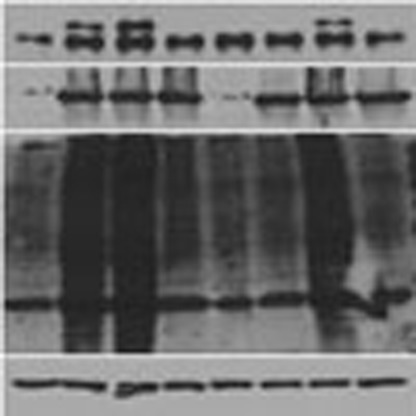

Macrophages play an important role in inflammation and host defense against various pathogens and therefore are an essential component of innate immune responses. Activated macrophages are defined as classically activated or M1 type and alternatively activated or M2 type^1^,^2^,^3^,^4^. In responses to Toll-like receptor (TLR) ligands and IFN-γ, macrophages undergo classical M1 activation, whereas macrophages will polarize to alternative M2 activation on stimulation with IL-4 and IL-13 (refs 5, 6). The M1 phenotype is characterized by the induction of proinflammatory mediators such as TNF-α, IL-6 and iNOS^2^,^5^. M1 cells promote Th1 and Th17 immune responses and contribute to a strong microbicidal and tumoricidal activity. In contrast, M2 macrophages are characterized by reduced responsiveness to TLR ligands and IFN-γ, resulting in the induction of low level of proinflammatory mediators and in the upregulation of arginase 1 (Arg1) and IL-10 (refs 2, 6). Although the molecular mechanisms that regulate M1 and M2 macrophage polarization are not fully understood, it appears that IRF5 is a key transcription factor for M1 macrophages while IRF4 is important for M2 macrophages^7^,^8^.

Increasing evidence suggests that M1 cells are involved in the pathogenesis of various autoimmune inflammatory diseases, including multiple sclerosis, rheumatoid arthritis, inflammatory bowel diseases and asthma^9^,^10^. Thus, a more complete understanding of the molecular mechanisms involved in the regulation of M1 innate immune responses should provide insights into the pathogenesis and treatment of these and possibly other inflammatory diseases. Although the activation programme for M1 macrophage differentiation has been well established, the intrinsic suppressive programme for M1 macrophage differentiation has not been fully understood.

Nitric oxide (NO), one of the smallest known bioactive products of mammalian cells, is critical to numerous physiological processes including host defense against pathogens, vasodilation and neurotransmission^11^,^12^. Three distinct isoforms of NO synthase have been identified, neuronal NOS (nNOS), inducible NOS (iNOS) and endothelial NOS (eNOS)^13^. nNOS and eNOS both are calcium-dependent and are primarily expressed in neurons and endothelial cells. Induction of iNOS varies depending on cell types and species^14^. The TLR ligands and inflammatory cytokines including IFN-γ can induce iNOS expression in many cell types. It is clear that NO is an important proinflammaotry cytotoxic mediator that defends the host against various pathogens by inactivating and destroying infectious agents^15^. iNOS is a signature molecule for M1 macrophages. Interestingly, NO also plays critical roles in immune suppression^16^,^17^. Previously, we and other groups reported that NO suppresses IL-12 production in dendritic cells and macrophages^18^, suggesting that NO may control the expression of molecules involved in the innate immune responses. In addition, iNOS-deficient mice are more susceptible than wild-type mice to the development of inflammatory diseases such as EAE^19^,^20^. Although it is clear that NO derived from iNOS is involved in the regulation of certain gene expression by innate immune cells, it is still not clear whether iNOS selectively regulates certain gene expressions in innate immune responses or iNOS modulates the differentiation of innate immune cells.

In the present study, we show that mice deficient in iNOS exhibited enhanced M1 macrophage polarization while exhibiting no significant effects on M2 macrophages. We demonstrated that L-NIL, an iNOS selective inhibitor, significantly enhanced M1 macrophage polarization in cell cultures from wild-type (WT) mice. Meanwhile, a NO donor, SNAP, suppressed M1 macrophage differentiation in WT and *iNOS*^*−/−*^ cell cultures. Furthermore, NO nitrated the tyrosine residues of IRF5 protein, resulting in the suppression of M1 macrophage polarization. Systems analyses demonstrate a mutually inhibitory circuit that dynamically fine-tunes the competitive expression of iNOS and IL-12 in macrophages. Transfer of iNOS-deficient macrophages into C57BL/6 mice lead to higher susceptibility to endotoxin shock. These findings suggest that NO plays a critical suppressive role in the control of M1 macrophage activation and highlight the importance of myeloid cell-derived iNOS in modulating M1 macrophage-mediated innate immune responses.

## Results

### iNOS deficiency enhances M1 macrophage differentiation

**Figure 1:**
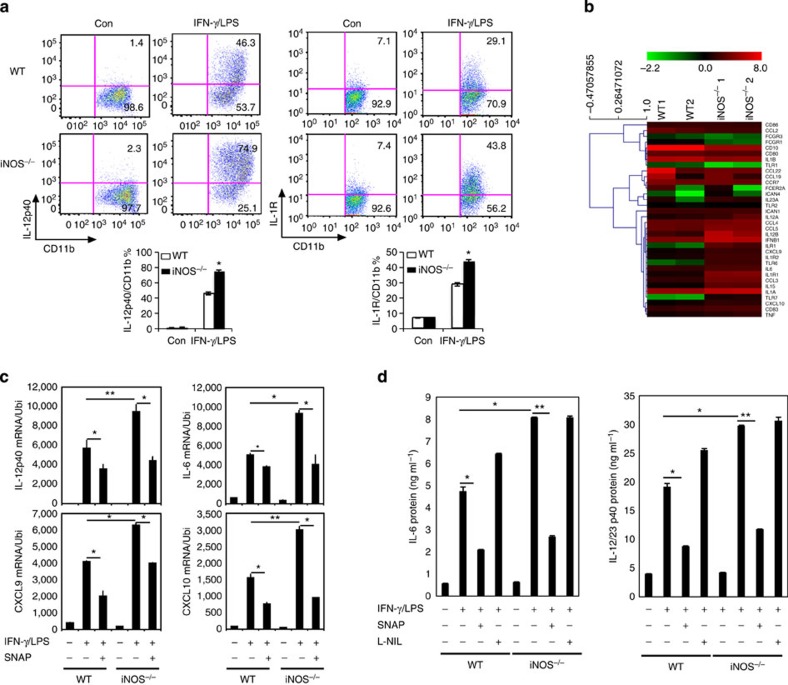
Enhanced M1 macrophage signature gene expression in iNOS-deficient mice. (**a**) BMDMs from wild type or *iNOS*^*−/−*^ mice were stimulated with IFN-γ (10 ng ml^−1^) plus LPS (200 ng ml^−1^) for 24 h, stained for intracellular IL-12 and surface IL-1R and analysed by flow cytometry. Representative FACS dot plots gated on CD11b^+^ cells and the percentages of IL-12-producing and IL-1R-positive CD11b^+^ cells are shown. Each bar represents mean±s.d. from three independent experiments, unpaired Student’s *t*-test, *P<0.05 versus WT cells. (**b**) The cells prepared in (**a**) were stimulated with IFN-γ (10 ng ml^−1^) plus LPS (200 ng ml^−1^) for 6 h and microarray experiment was performed for the analysis of M1 macrophage gene mRNA expression. (**c**) The cells prepared in (**b**) in the presence of SNAP (500 μM) were stimulated with IFN-γ (10 ng ml^−1^) plus LPS (200 ng ml^−1^) for 6 h, and mRNA expression of indicated genes was determined by qPCR. The data shown were normalized to levels of ubiquitin expression. Each bar represents mean±s.d. from three independent experiments, one-way ANOVA with a Bonferroni correction, *P<0.05; **P<0.01. (**d**) The cells prepared in (**a**) in presence of SNAP (500 μM) or L-NIL (40 μM) were stimulated with IFN-γ (10 ng ml^−1^) plus LPS (200 ng ml^−1^) for 24 h and the supernatants were analysed for IL-12 and IL-6 by ELISA. Each bar represents mean±s.d. from three independent experiments, one-way ANOVA with a Bonferroni correction, *P<0.05; **P<0.01.

To investigate the function of nitric oxide (NO) in macrophage cell differentiation, we first assessed the characteristics of macrophage development in iNOS-deficient mice. Bone marrow cells from *iNOS*^*−/−*^ or WT control mice were incubated with GM-CSF (10 ng ml^−1^) or M-CSF (10 ng ml^−1^) *in vitro* for 7 days. The cells were activated with LPS (200 ng ml^−1^) plus IFN-γ (10 ng ml^−1^) for M1 macrophage differentiation and examined for the percentages of IL-12 p40-producing cells by intracellular staining and IL-1R expressing cells using flow cytometry. Notably, the frequency of IL-12-producing cells or IL-1R-expressing cells generated from *iNOS*^*−/−*^ macrophage cultures was significantly greater than that of cells from WT cultures (Fig. 1a). Next, we performed micro-array experiment to determine M1 macrophage marker gene expression. Interestingly, the mRNA expression of the M1 marker genes including IL-12A, IL-6, TNFα, IL-1R, CXCL9 and CXCL10 was significantly enhanced in *iNOS*^*−/−*^ M1 macrophages compared with that of WT cells (Fig. 1b). To confirm the micro-array results, we performed qPCR experiments and found that mRNA expression of IL-12A, IL-6, CXCL9 and CXCL10 was indeed increased (Fig. 1c, Supplementary Fig. 1). These observations correlated with enhanced IL-12A and IL-6 secretion by *iNOS*^*−/−*^ M1 macrophages as determined by ELISA (Fig. 1d). To rule out the possibility that the enhanced M1 cell differentiation was due to abnormal myeloid cell development, we analysed myeloid cell population from spleens and bone marrows of WT and *iNOS*^*−/−*^ mice (Supplementary Fig. 2). In contrast to the striking effect of iNOS deficiency on M1 macrophage differentiation, M2 macrophage signature gene expression was not significantly affected in *iNOS*^*−/−*^ macrophage cultures (Supplementary Fig. 3). In addition, we analysed whether eNOS or nNOS deficiency affects M1 macrophage differentiation. Bone marrow cells from *eNOS*^*−/−*^, *nNOS*^*−/−*^, or WT control mice were incubated with GM-CSF (10 ng ml^−1^) *in vitro* for 7 days. The cells were then either activated with LPS (200 ng ml^−1^) plus IFN-γ (10 ng ml^−1^) for M1 macrophage differentiation. The results showed that neither eNOS deficiency nor nNOS deficiency affects M1 macrophage differentiation (Supplementary Figs 4 and 5). Taken together, these results indicate that M1 macrophage cell differentiation is enhanced in macrophages deficient in iNOS, suggesting that NO plays a negative role in M1 macrophage differentiation.

To investigate whether the enhancement of M1 macrophage differentiation was the result of alteration of IL-10 production in iNOS-deficient mice, we examined IL-10 mRNA expression in WT and *iNOS*^*−/−*^M1 macrophages. It seems that IL-10 mRNA expression was comparable in WT and *iNOS*^*−/−*^M1 macrophages and NO donor SNAP did inhibit M1 macrophage signature gene expression in *IL-10*^*−/−*^ mice (Supplementary Fig. 6). Thus, the enhanced M1 macrophage differentiation in *iNOS*^*−/−*^ mice was not due to the alterations of IL-10 expression.

**Figure 2:**
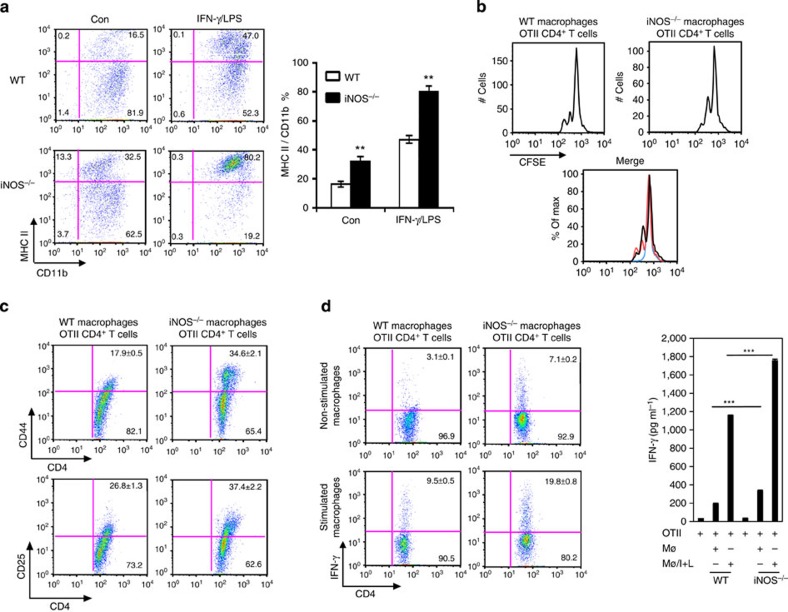
*iNOS*^*−/−*^ macrophages induce enhanced CD4^+^ T-cell activation. (**a**) BMDMs from WT or *iNOS*^*−/−*^ mice were stimulated with IFN-γ (10 ng ml^−1^) plus LPS (200 ng ml^−1^) for 24 h, stained for surface MHC II and analyzed by flow cytometry. Representative FACS dot plots gated on CD11b^+^ cells and the percentage of MHC II-positive CD11b^+^ cells are shown. Each bar represents mean±s.d. from three independent experiments, unpaired Student’s *t*-test, **P<0.01 versus WT cells. (**b**) CD4^+^ T cells from spleens and lymph nodes of OTII mice were prepared and the cells were labelled with CFSE. The labelled cells were incubated with WT or *iNOS*^*−/−*^ BMDMs for 3 days. T-cell proliferation was analysed by flow cytometry. (**c**) CD4^+^ T cells from spleens and lymph nodes of OTII mice were prepared and the cells were incubated with WT or *iNOS*^*−/−*^ bone marrow-derived macrophages for 3 days. The cells were stained for CD44 and CD25 and analysed by flow cytometry. Representative FACS dot plots gated on CD11b^+^ cells and the percentages of CD44- and CD25-positive CD11b^+^ cells are shown. (**d**) CD4^+^ T cells from spleens and lymph nodes of OTII mice were prepared, and the cells were incubated with WT (unstimulated or stimulated with IFN-γ and LPS) or *iNOS*^*−/−*^ (unstimulated or stimulated with IFN-γ and LPS) BMDMs for 3 days. The cells were stained for intracellular IFN-γ and analysed by flow cytometry. Representative FACS dot plots gated on CD4^+^ cells and the percentages of IFN-γ-producing CD4^+^ cells are shown. The supernatants were analysed for IFN-γ by ELISA. Each bar represents mean±s.d. from three independent experiments, unpaired Student’s *t*-test, ***P<0.001.

To understand how *iNOS*^*−/−*^macrophages affect T-cell activation, we first analysed MHC II expression in iNOS-deficient macrophages. Bone marrow cells from *iNOS*^*−/−*^ or WT control mice were incubated with GM-CSF (10 ng ml^−1^) *in vitro* for 7 days. The cells were then activated with LPS (200 ng ml^−1^) plus IFN-γ (10 ng ml^−1^) for M1 macrophage differentiation and examined for MHCII-positive cells. The results showed that the percentage of MHCII-positive cells was significantly higher in iNOS-deficient mice (Fig. 2a). In addition, SNAP significantly decreased the percentage of MHCII-positive cells while L-NIL clearly increased the percentage of MHCII-positive cells in WT cell cultures (Supplementary Fig. 7). Next, we co-cultured WT or *iNOS*^*−/−*^ macrophages with OTII CD4^+^ T cells. CFSE dilution assay indicated that T-cell proliferation was comparable in cultures either with WT or *iNOS*^*−/−*^ macrophages (Fig. 2b), suggesting that iNOS deficiency in macrophage did not alter T-cell proliferation. However, the activation markers including CD44 and CD25 were significantly increased in CD4^+^ T cells co-cultured with iNOS-deficient macrophages (Fig. 2c). Furthermore, the population of IFN-γ-producing T cells and production of IFN-γ was significantly enhanced in cultures with iNOS-deficient macrophages (Fig. 2d). Taken together, the results suggest that *iNOS*^*−/−*^ macrophages induce strong T-cell activation.

### iNOS expression is modulated by NO in macrophages

**Figure 3:**
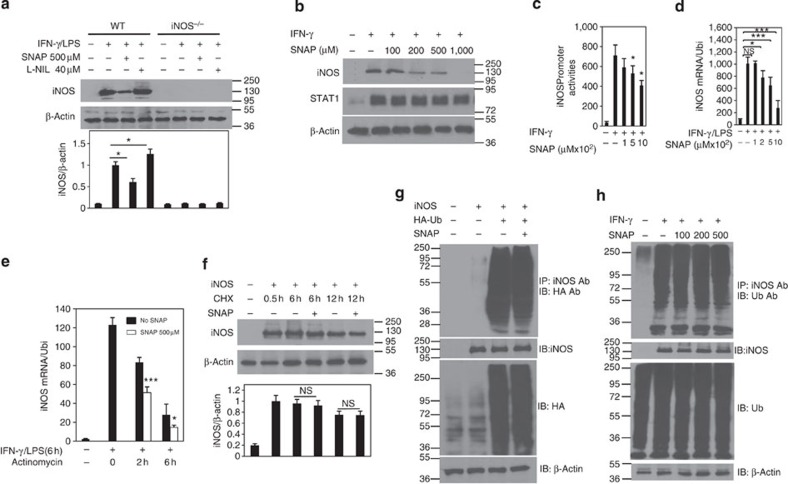
NO suppresses iNOS expression in macrophages. (**a**) BMDMs from WT and *iNOS*^*−/−*^ mice were stimulated with IFN-γ (10 ng ml^−1^) and LPS (200 ng ml^−1^) in the presence of SNAP (500 μM) or L-NIL (40 μM) overnight. The cell lysates were prepared and western blotting was performed for the analysis of iNOS protein expression. β-Actin expression was used as a control. Each bar represents mean±s.d. from three independent experiments, one-way ANOVA with a Bonferroni correction, *P<0.05. (**b**) Macrophage cell line RAW 264.7 cells were stimulated with IFN-γ (10 ng ml^−1^) in the presence of SNAP at different concentrations (100, 200, 500 and 1,000 μM) overnight. The cell lysates were prepared and western blotting was performed for the analysis of iNOS protein expression. β-Actin expression was used as a control. (**c**) RAW 264.7 cells were transfected with iNOS promoter luciferase plasmid overnight and then the cells were stimulated with IFN-γ (10 ng ml^−1^) in the presence of SNAP at different concentrations (100, 500 and 1,000 μM) for 12 h. Luciferase assays were performed and luciferase activities were normalized to β-galactosidase activity. Each bar represents mean±s.d. from three independent experiments, unpaired Student’s *t*-test, *P<0.05 versus IFN-γ stimulation only. (**d**) BMDMs from WT were stimulated with IFN-γ (10 ng ml^−1^) and LPS (200 ng ml^−1^) in the presence of SNAP at different concentrations (100, 200, 500 and 1,000 μM) for 6 h. Total cellular RNA was extracted, and qPCR was performed for the analysis of iNOS mRNA expression. Each bar represents mean±s.d. from three independent experiments, unpaired Student’s *t*-test, *P<0.05 and ***P<0.001 versus IFN-γ and LPS (200 ng ml^−1^) stimulation only. (**e**) BMDMs from C57BL/6 mice were stimulated with IFN-γ (10 ng ml^−1^) and LPS (200 ng ml^−1^) for 6 h, and the cells were then washed with PBS for four times. The cells were incubated with new medium in the presence of actinomycin for 30 min and then were treated with or without SNAP for 2 or 6 h. qPCR was performed for the analysis of iNOS mRNA expression. Each bar represents mean±s.d. from three independent experiments, unpaired Student’s *t*-test, *P<0.05 and ***P<0.001, versus no addition of SNAP. (**f**) The 293T cells were transfected with iNOS overexpression plasmid for 36 h. The cells were treated with CHX (10 μM) for 30 min, and the cells were then treated with or without SNAP for different time intervals. Western blotting was performed for the analysis of iNOS protein expression. Each bar represents mean±s.d. from three independent experiments, unpaired Student’s *t*-test, not significant (NS) (**g**) The 293T cells were transfected with iNOS overexpression plasmid and HA-tagged ubiquitin overexpression plasmid for 36 h. The cell lysates were immunoprecipitated with an anti-iNOS antibody and immunoblotted with an anti-HA antibody. (**h**) RAW 264.7 cells were stimulated with IFN-γ (10 ng ml^−1^) overnight and the cells were washed five times with fresh medium. The cells were then incubated with new medium in the presence of SNAP at different concentrations for 12 h. The cell lysates were immunoprecipitated with an anti-iNOS antibody and immunoblotted with an anti-ubiquitin antibody. All experiments were repeated three times with similar results.

As iNOS expression is an important marker for M1 macrophage differentiation, we tested how NO derived from iNOS regulates the expression of iNOS itself. Bone marrow-derived macrophages from WT and *iNOS*^*−/−*^ mice were activated by LPS plus IFN-γ in the presence of SNAP (a NO donor) or L-NIL (an iNOS specific inhibitor) for 24 h. Western blotting showed that iNOS protein was indeed induced in WT M1 macrophages but not in *iNOS*^*−/−*^ cells (Fig. 3a). Interestingly, SNAP significantly reduced iNOS protein expression while L-NIL clearly enhanced iNOS protein expression (Fig. 3a,b). To understand the effect of NO on the regulation of iNOS at the transcriptional level, we transfected iNOS promoter luciferase reporter plasmid into RAW264.7 cells for 18 h and the cells were then activated with IFN-γ in the presence of SNAP for additional 12 h. We found that SNAP dose dependently suppressed iNOS promoter activation (Fig. 3c). In addition, SNAP significantly suppressed iNOS mRNA expression (Fig. 3d). Furthermore, we found that SNAP impaired iNOS mRNA stability (Fig. 3e). These results suggest that NO regulates iNOS expression at the transcriptional level. To further explore whether NO affects iNOS protein posttranslational modification, we transfected iNOS overexpression plasmid into 293T cells for 36 h and the cells were then treated with SNAP (500 μM) in the presence of cycloheximide (10 μM) for different time intervals. The results showed that addition of SNAP had no significant effect on iNOS protein stability (Fig. 3f). Furthermore, co-immunoprecipitation experiment showed SNAP did not affect iNOS ubiquitination. To confirm these results, we stimulated macrophage RAW264.7 cells with IFN-γ (10 ng ml^−1^) for 24 h. Afterwards the cells were washed five times with fresh medium and incubated with fresh medium in the presence of SNAP for 12 h. Similarly, addition of SNAP had no clear effect on iNOS protein stability and iNOS ubiquitination (Fig. 3g,h). Thus, NO significantly suppresses iNOS expression at the transcriptional level and had no noticeable effects on iNOS protein stability.

### NO suppresses IRF5 DNA binding activity in macrophages

**Figure 4:**
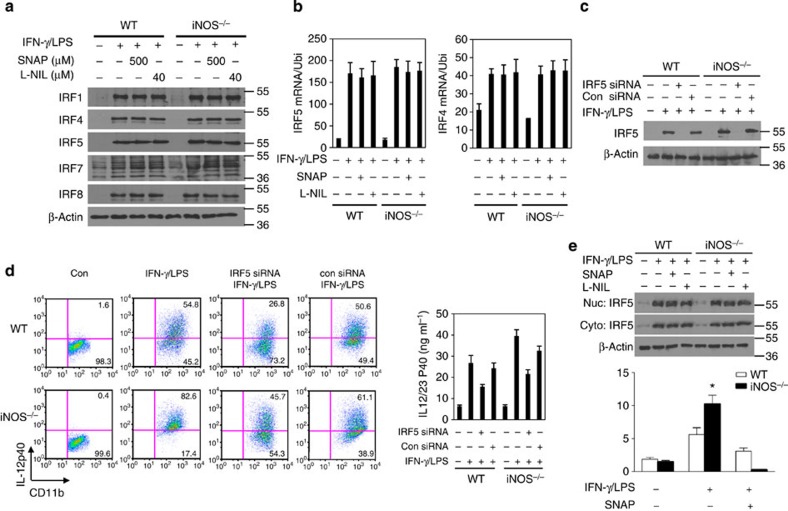
NO suppresses IRF5 DNA binding activity. (**a**) BMDMs from WT and *iNOS*^*−/−*^ mice were stimulated with IFN-γ (10 ng ml^−1^) and LPS (200 ng ml^−1^) in the presence of SNAP (500 μM) or L-NIL (40 μM) overnight. The cell lysates were prepared and western blotting was performed for the analysis of protein expression of indicated genes. (**b**) BMDMs were activated with IFN-γ (10 ng ml^−1^) and LPS (200 ng ml^−1^) in the presence of SNAP (500 μM) for 6 h and total cellular RNA was extracted. qPCR was performed for the analysis of mRNA expression of IRF5 and IRF4. (**c**) BMDMs were transfected with IRF5 siRNA or control siRNA, and the cells were then stimulated with IFN-γ (10 ng ml^−1^) and LPS (200 ng ml^−1^) overnight. The cell lysates were prepared and western blotting was performed for the analysis of IRF5 protein expression. (**d**) BMDMs were transfected with IRF5 siRNA or control siRNA, and the cells were then stimulated with IFN-γ (10 ng ml^−1^) and LPS (200 ng ml^−1^) overnight. The cells were stained for intracellular IL-12 and analysed by flow cytometry. Representative FACS dot plots gated on CD11b^+^ cells, and the percentage of IL-12-producing CD11b^+^ cells is shown. IL-12/23 p40 production was determined by ELISA. Each bar represents mean±s.d. from three independent experiments. (**e**) BMDMs from WT and *iNOS*^*−/−*^ mice were stimulated with IFN-γ (10 ng ml^−1^) and LPS (200 ng ml^−1^) in the presence of SNAP (500 μM) or L-NIL (40 μM) overnight. The cytosolic fraction and nuclear fraction of protein was prepared, and western blotting was performed for the analysis of IRF5 protein expression (upper panel). BMDMs from WT and *iNOS*^*−/−*^ mice were stimulated with IFN-γ (10 ng ml^−1^) and LPS (200 ng ml^−1^) in the presence of SNAP (500 μM) or L-NIL overnight, followed by ChIP assay. Three micrograms of an anti-IRF5 antibody or isotype-matched IgG as control antibody were used in the immunoprecipitation step. PCR was used to quantify the amount of precipitated DNA with primers flanking the IRF5-binding site of the IL-12 promoter region (lower panel). Each bar represents mean±s.d. from three independent experiments, unpaired Student’s *t*-test, *P<0.05, versus WT cells.

**Figure 5:**
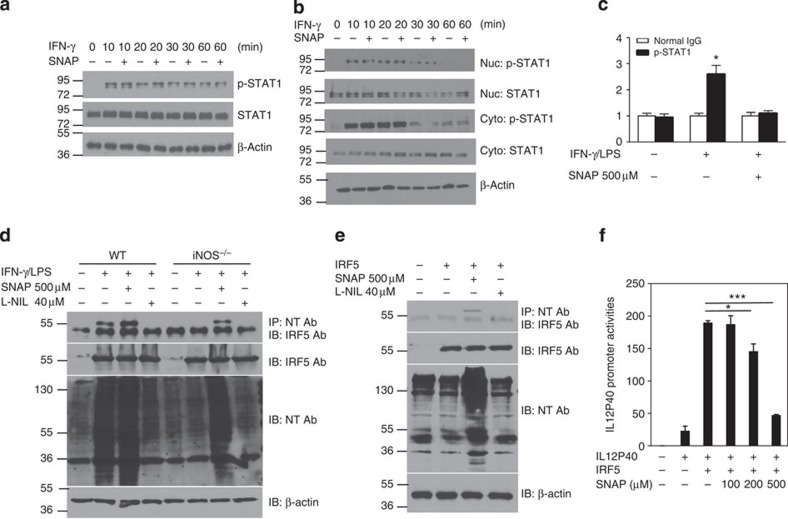
NO induces nitration of tyrosine residues of IRF5 protein in macrophages. (**a**) RAW264.7 macrophages were pretreated with SNAP (500 μM) for 30 min and the cells were then activated with IFN-γ (10 ng ml^−1^) for various time intervals (10, 20, 30 and 60 min). Western blotting was performed for the analysis of STAT1 phosphorylation. β-Actin was used as a control. (**b**) RAW264.7 macrophages were pretreated with SNAP (500 μM) for 30 min, and the cells were then activated with IFN-γ (10 ng ml^−1^) for various time intervals (10, 20, 30 and 60 min). The cytosolic fraction and nuclear fraction of protein was prepared, and western blotting was performed for the analysis of phosphorylated STAT1 and STAT1 protein. (**c**) BMDMs from WT mice were stimulated with IFN-γ (10 ng ml^−1^) and LPS (200 ng ml^−1^) in the presence of SNAP (500 μM) for 6 h, followed by ChIP assay. Three micrograms of an anti- phosphorylated STAT1 antibody or isotype-matched IgG as control antibody was used in the immunoprecipitation step. PCR was used to quantify the amount of precipitated DNA with primers flanking the STAT1-binding site of the iNOS promoter region. Each bar represents mean±s.d. from three independent experiments, unpaired Student’s *t*-test, *P<0.05, versus normal IgG. (**d**) BMDMs from WT and *iNOS*^*−/−*^ mice were stimulated with IFN-γ (10 ng ml^−1^) and LPS (200 ng ml^−1^) in the presence of SNAP (500 μM) or L-NIL (40 μM) for 24 h. The cell lysates were then immunoprecipitated with an anti-nitrotyrosine antibody and blotted with an anti-IRF5 antibody. Data are representative of three independent experiments. (**e**) The 293T cells were transfected with IRF5 plasmid for 40 h in the presence of SNAP (500 μM) or L-NIL (40 μM). Cell lysates were immunoprecipitated with an anti-nitrotyrosine antibody and immunoblotted with an anti-IRF5 antibody. (**f**) The 293T cells were cotransfected with an IL-12 promoter reporter construct and an IRF5 plasmid in the presence of SNAP (100, 200 and 500 μM) for 30 h. Luciferase assays were performed and luciferase activities were normalized to β-galactosidase activity. Each bar represents mean±s.d. from three independent experiments, unpaired Student’s *t*-test, *P<0.05 and ***P<0.001 versus IRF5 plasmid only.

The above findings prompted us to probe the molecular basis for NO control of M1 macrophage differentiation. Since many studies have demonstrated that IRF5 plays a critical role in M1 macrophage differentiation both *in vitro* and *in vivo*^7^, we asked whether NO might affect IRF5 expression, resulting in the control of M1 macrophage differentiation. As such, we first activated bone marrow-derived macrophages from WT and *iNOS*^*−/−*^ mice with LPS plus IFN-γ for 6 h for real-time PCR or 20 h (protein) for western blotting. The results showed that IRF5 mRNA and protein expression was similar in *iNOS*^*−/−*^ M1 macrophage compared with WT cells (Fig. 4a). In addition, the protein expression of other transcription factors including IRF4 protein expression was comparable between *iNOS*^*−/−*^ and WT M1 macrophages (Fig. 4b). Furthermore, qPCR experiments showed that NO donor SNAP and iNOS-specific inhibitor L-NIL had any noticeable effect on IRF5 mRNA expression (Fig. 4b). To investigate whether IRF5 is responsible for enhancing M1 macrophage differentiation in *iNOS*^*−/−*^ mice, bone marrow-derived macrophages from WT and *iNOS*^*−/−*^ mice were transfected with IRF5 siRNA or control siRNA and the cells were then activated with LPS plus IFN-γ (Fig. 4c). Flow cytometry analysis showed that the percentage of IL-12-positive cells was significantly reduced in IRF5-knockdown cells of WT and *iNOS*^*−/−*^ mice (Fig. 4d). In addition, IRF5 knockdown clearly reduced IL-12/23 p40 cytokine release in WT and *iNOS*^*−/−*^ cell culture (Fig. 4d). These results suggest that IRF5 is involved in the regulation M1 macrophage differentiation in *iNOS*^*−/−*^ mice. Next we wanted to investigate how iNOS affects IRF5-targeted M1 macrophage signature genes. Bone marrow cells from *iNOS*^*−/−*^ or WT control mice were incubated with GM-CSF (10 ng ml^−1^) *in vitro* for 7 days and then the cells were activated with LPS (200 ng ml^−1^) plus IFN-γ (10 ng ml^−1^) for M1 macrophage differentiation in the presence of SNAP (500 μM) for 20 h. iNOS deficiency has no effects on IRF5 translocation (Fig. 4e). However, chromatin immunoprecipitation (ChIP) experiments showed the DNA binding activity of IRF5 to the promoter region of *IL-12 p40* gene was significantly increased in *iNOS*^*−/−*^ M1 macrophages while SNAP greatly suppressed IRF5 DNA binding activity (Fig. 4e). In addition, we also analysed the effects of NO on the expression of STAT1, a transcription factor in IFN-γ signalling cascade. Macrophage RAW264.7 cells were pretreated with SNAP (500 μM) for 30 min and then the cells were activated with IFN-γ for various time intervals (10, 20 and 60 min). Western blotting showed that SNAP had no effect on the phosphorylation of STAT1 (Fig. 5a). In addition, SNAP did not affect STAT1 translocation (Fig. 5b). The DNA binding activity of STAT1 to the GAS element of iNOS promoter was significantly induced by IFN-γ/LPS in WT M1 macrophages and SNAP clearly suppressed STAT1 DNA binding activity (Fig. 5c).

**Figure 6:**
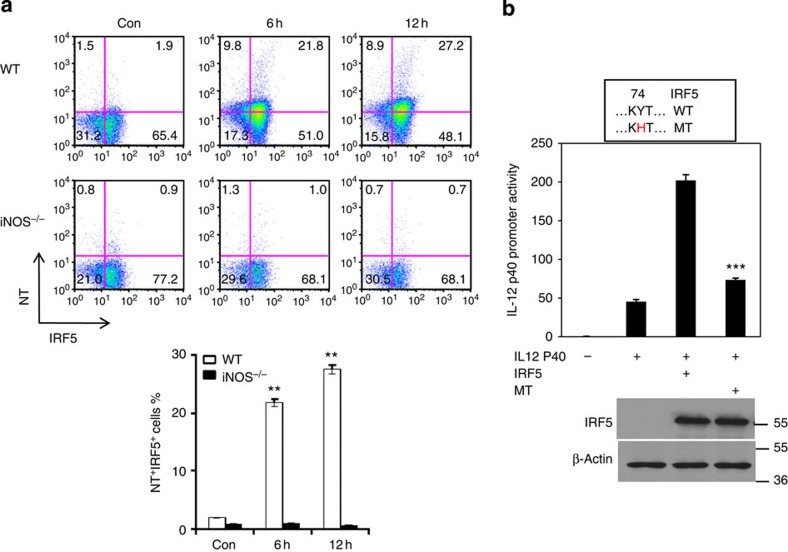
NO induces nitration of tyrosine residues of IRF5 protein in macrophages. (**a**) WT or *iNOS*^*−/−*^ mice were injected (i.p.) with LPS (200 μg/mouse) for 6 or 12 h. Mice were then killed and spleen cells were prepared. The cells were stained for IRF5 and nitrotyrosine and analysed by flow cytometry. Representative FACS dot plots gated on CD11b^+^ cells, and the percentages of IRF5 and nitrotyrosine-positive CD11b^+^ cells are shown. Each bar represents mean±s.d. from three independent experiments, unpaired Student’s *t*-test, **P<0.01, versus *iNOS*^*−/−*^ cells. (**b**) The 293T cells were transfected with an IL-12 p40 promoter reporter construct and wild-type IRF5 or mutant IRF5Y74H plasmids for 30 h. Luciferase assays were performed, and luciferase activities were normalized to β-galactosidase activity. In addition, IRF5 protein expression was analysed by western blotting. Each bar represents mean±s.d. from three independent experiments, unpaired Student’s *t*-test, ***P<0.001, versus cells transfected with WT IRF5 plasmid.

The data above showed that iNOS had no effect on IRF5 expression but significantly affected IRF5 DNA binding activity. We then proceeded to analyse whether NO modulates posttranslational modification of IRF5 protein. The amino-acid residue sequence in IRF5 protein has tyrosine residues, which may be subject to nitration induced by NO. To investigate this possibility, we activated bone marrow-derived macrophages from WT and *iNOS*^*−/−*^ mice with LPS plus IFN-γ for 20 h, and the cell lysates were immunoprecipitated with an anti-nitrotyrosine antibody and immunoblotted with an IRF5 antibody. The results showed that IRF5 protein was nitrated in macrophages activated with LPS plus IFN-γ in WT macrophages but not in *iNOS*^*−/−*^ cells (Fig. 5d). To confirm these results, 293T cells were transfected with IRF5 overexpression plasmid in the presence of SNAP or L-NIL for 40 h. Cell lysates were immunoprecipitated with an anti-nitrotyosine antibody and immunoblotted with an anti-IRF5 antibody. SNAP treatment clearly induced tyrosine nitration of IRF5 (Fig. 5e), suggesting that NO-induced alterations of tyrosine residues may affect IRF5 activation. To examine the effect of NO on IRF5 at the functional level, we co-transfected an IL-12 promoter reporter and IRF5 expression plasmids into 293T cells in the presence of various doses of SNAP for 36 h and analysed them for IL-12 promoter activation. The data showed that SNAP suppressed IRF5-mediated IL-12 promoter activation in a dose-dependent manner (Fig. 5f). To investigate whether IRF5 is nitrated *in vivo*, we injected (i.p.) LPS (200 μg mouse^−1^) into WT and iNOS^*−/−*^ mice. The results clearly showed that IRF5 was nitrated in WT mice but not in iNOS^*−/−*^ mice (Fig. 6a). Bioinformatic analysis suggests that tyrosine residue Tyr74 in IRF5 protein is most likely to be subject to nitration. We found that mutating this tyrosine residue significantly impaired IRF5-mediated IL-12 p40 promoter activation, suggesting that Tyr74 is critical for IRF5 transcriptional function (Fig. 6b). In addition, mutating Tyr104 also impaired IRF5-mediated IL-12 p40 promoter activation (Supplementary Fig. 8). Taken together, these results suggest that NO suppresses M1 macrophage differentiation at the transcriptional level by nitration of tyrosine residues in IRF5.

### Dynamic modulation of IL-12 expression by iNOS in macrophages

**Figure 7:**
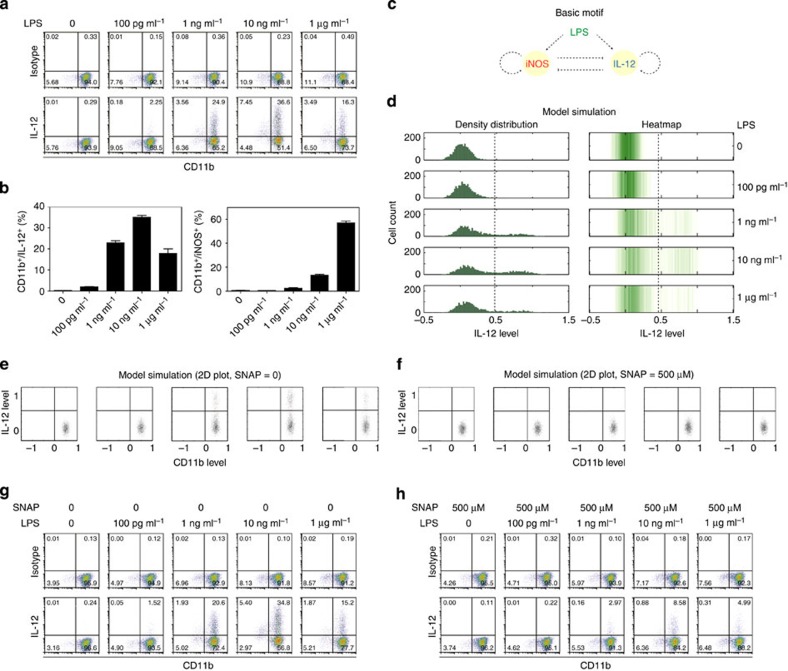
Dynamic modulation of IL-12 expression by iNOS in macrophages. (**a**) BMDMs were treated with different doses of LPS overnight, and then re-stimulated with PMA (20 ng ml^−1^) and ionomycin (1 μg ml^−1^) in the presence of monensin (2 μM) for 4 h. The surface expression of CD11b and intracellular expression of IL-12 was examined by flow cytometry. The quadrant gates were created based on the staining of isotype matched antibodies. (**b**) Quantitation of the expression levels of IL-12 (left panel) and iNOS (right panel) by varying dosages of LPS. (**c**) The basic motif of iNOS and IL-12 competition. (**d**) The converted heatmaps of the distribution of IL-12 expression. In agreement with experimental observation, the percentages of IL-12-positive cells have a biphasic behaviour under LPS treatment. (**e**) Model predicts that NO donor can attenuate the biphasic dynamics of IL-12 under treatment of increasing LPS. (**f**) Model simulation of IL-12 expression when 500 μM SNAP (a NO donor) was applied. A total number of 2,000 cells are simulated with different LPS level (0, 0.1, 1, 10 and 1,000 ng ml^−1^). (**g**,**h**) BMDMs were treated overnight with LPS in the presence of vehicle control (**g**) of SNAP (**h**). IL-12 expression was analysed within the CD11b^+^ population. The quadrant gates were created based in the staining of isotype matched control antibodies. The data are representative of three similar experiments.

We next set up experiments to test the dynamic IL-12 expression in macrophages. To study this, we used LPS alone instead of LPS plus IFN-γ as the stimulant. The rationale is that LPS alone will not restrict macrophages to a fixed phenotype. Rather, we and other observed that varying dosages of LPS will induce dynamic modulation of both pro- and anti-inflammatory mediators^21^,^22^,^23^,^24^. Bone marrow macrophages from WT mice were treated with varying dosages of LPS (100 pg ml^−1^, 1, 10, 1,000 ng ml^−1^) overnight. Intracellular IL-12 was stained with a specific fluorescence-conjugated antibody and stained macrophages were analysed by flow cytometry. We observed that the expression of IL-12 was dynamically modulated based on the concentration of LPS (Fig. 7a). Super low-dose LPS moderately elevated the IL-12-expressing population. The induction of IL-12 reached the maximum at moderate LPS concentration (1–10 ng ml^−1^). As the concentration of LPS further rises, the trend reversed. The population of IL-12-expressing cells dropped in cells treated with 1 μg ml^−1^ LPS. In contrast, the expression of iNOS was not induced by 100 pg ml^−1^ LPS, but was only markedly induced with higher-dose LPS (Fig. 7b). In addition, we observed that IL-12 production was increased with the increasing concentrations of LPS up to 1 μg ml^−1^ in iNOS-deficient macrophages (Supplementary Fig. 9), and NO donor suppressed IL-12 production (Supplementary Fig. 9). On the basis of our above-described mechanistic studies, we distilled the competitive nature of iNOS and IL-12 expression into a simple computational motif as shown in Fig. 7c. In this circuit, LPS is activating two competing signalling programmes that differentially induce either iNOS or IL-12. In the meantime, these two programmes are mutually inhibitory. The signal strength leading to iNOS or IL-12 expression varies based on LPS concentration. Computational simulation of this motif gave rise to similar dynamic expression profiles of IL-12 (Fig. 7d).

On the basis of this computational analysis, we further predict that the addition of NO donor SNAP would destroy the dynamic induction of IL-12 by varying dosages of LPS (Fig. 7e,f). Indeed, our experimental data confirmed this prediction and demonstrated that SNAP treatment significantly reduced the IL-12-expressing population induced by LPS (Fig. 7g,h).

### iNOS regulates M1 macrophage differentiation *in vivo*

**Figure 8:**
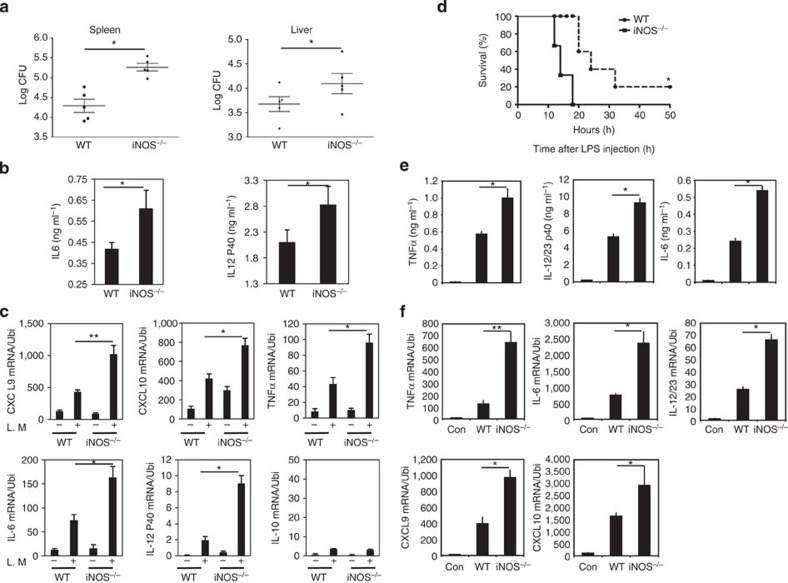
iNOS deficiency promotes the M1 macrophage differentiation *in vivo*. WT or *iNOS*^*−/−*^ mice were injected (i.v) with 2 × 10^4^ CFU of viable *L. monocytogenes* for 2 days. (**a**) Mice were killed and spleens and livers were removed. Spleens and livers were homogenized and viable bacteria were numerated by the pour plate method after serial dilution. Data represent mean±s.d. (*n*=5), unpaired Student’s *t*-test, *P<0.05. (**b**) The sera level of IL-6 and IL-12p40 was determined by ELISA. Data represent mean±s.d. (*n*=5), unpaired Student’s *t*-test, *P<0.05. (**c**) Total cellular RNA was extracted from spleens, and qPCR was performed for the analysis of mRNA expression of indicated genes. The data are representative of two similar experiments, unpaired Student’s *t*-test, *P<0.05, **P<0.01. (**d**) C57Bl/6 mice were transferred with 2 × 10^6^ WT macrophages or *iNOS*^*−/−*^ macrophages for 24 h, and the recipient mice were then challenged with LPS (800 μg mouse^−1^). The survival of mice was observed (*n*=8 in each group). Kaplan–Meier method was used to estimate overall survival and the Log-rank test was applied to determine the difference of survival rate; *P<0.05. (**e**) The recipient mice were challenged with LPS (800 μg mouse^−1^) for 6 h, and sera level of cytokines was determined by ELISA, unpaired Student’s *t*-test, *P<0.05. (**f**) The recipient mice were challenged with LPS (800 μg mouse^−1^) for 6 h and spleens were removed. Total cellular RNA was extracted, and qPCR was performed for the analysis of mRNA expression of indicated genes. The data are representative of two similar experiments, unpaired Student’s *t*-test, *P<0.05, **P<0.01.

It is well-established that iNOS-derived NO plays an important role in host defense against bacterial infection by killing bacteria directly. Accumulating evidence indicates that iNOS-deficient mice are susceptible to bacterial infection. However, an important question remains unaddressed yet: what is the state of macrophage differentiation in iNOS-deficient mice during bacterial infection? To answer this question, we injected i.v. into WT and *iNOS*^*−/−*^ mice *L. monocytogenes* (2 × 10^4^ CFU per mouse) for 2 days and mice were then killed. Bacterial loads in the spleens and livers of iNOS-deficient mice were significantly increased compared with WT mice (Fig. 8a). Interestingly, the production of M1 macrophage signature molecule TNFα and IL-12 in the sera was clearly enhanced in *iNOS*^*−/−*^ mice (Fig. 8b). In addition, mRNA expression of M1 macrophage signature genes was also increased in *iNOS*^*−/−*^ mice after *L. monocytogenes* infection (Fig. 8c, Supplementary Fig. 10). These results indicate that M1 macrophage differentiation was actually enhanced in *iNOS*^*−/−*^ mice, although *iNOS*^*−/−*^ mice are susceptible to *L. monocytogenes* infection. To confirm this result, we prepared thioglycollate-elicted macrophages or BMDMs from WT and *iNOS*^*−/−*^ mice and transferred the cells into WT mice. Twenty-fours later, mice were challenged with LPS. Consequently, susceptibility and the expression of M1 macrophage signature molecules were observed. The results showed that mice transferred with *iNOS*^*−/−*^ macrophages were more susceptible to endotoxin shock (Fig. 8d, Supplementary Fig. 10a), and the production and expression of M1 macrophage signature molecules were significantly enhanced in mice transferred with *iNOS*^*−/−*^ macrophages (Fig. 8e,f, Supplementary Fig. 11b,c). Thus, iNOS deficiency in macrophages promotes M1 macrophage differentiation in both infection and endotoxin shock models, suggesting that iNOS expressed in macrophages may play a negative role in the regulation of innate immune response.

To further investigate the role of iNOS in macrophage function *in vivo*, we extended our studies to include a tumour cell inoculation model. C57Bl/6 mice were inoculated with melanoma cell line B16 cells. Then mice were treated either with PBS or iNOS specific inhibitor L-NIL. Interestingly, L-NIL treatment significantly decreased the tumour size in tumour-bearing mice (Supplementary Fig. 12). In addition, tumour-infiltrating cell analysis showed that L-NIL treatment significantly increased the percentage of M1 macrophages in the tumour microenvironment (Supplementary Fig. 12). Thus, iNOS inhibition in tumour microenvironment facilitates M1 macrophage differentiation, resulting in the decrease of tumour size, further confirming that NO negatively regulates M1 macrophage differentiation *in vivo*.

## Discussion

Classically activated macrophages (M1) play an important role in host defense against pathogen infection and are also involved in the pathogenesis of autoimmune and inflammatory diseases. Therefore, understanding the intrinsic modulating programmes for M1 macrophage differentiation will help us understand the mechanisms for the control of innate immune responses and dissect the mechanism involved in the development of human inflammatory diseases. In the present study, we demonstrate that iNOS-deficient mice displayed enhanced M1 macrophage differentiation but without major effects on alternatively activated macrophages (M2). Addition of N6-(1-iminoethyl)-L-lysine dihydrochloride (L-NIL), the iNOS inhibitor, significantly enhanced M1 macrophage differentiation, and *S*-nitroso-*N*-acetylpenicillamine (SNAP), the NO donor, suppressed M1 macrophage differentiation. NO derived from iNOS-mediated nitration of tyrosine residues in an IRF5 protein leading to the suppression of IRF5-targetted M1 macrophage signature gene activation, indicating that NO regulates macrophage differentiation by modulating IRF5. Finally, studies of an experimental model of endotoxin shock showed that iNOS deficiency results in more severe inflammation with an enhanced M1 macrophage activation phenotype. These results suggest that iNOS derived from macrophages selectively modulates M1 macrophage differentiation.

iNOS is expressed in different cell types including, macrophages, dendritic cells, NK cells and primary tumour cells^11^,^25^. NO derived from iNOS in macrophages and other innate immune cells is pro-inflammatory and an essential component of host defenses against various pathogens including bacteria, parasites and viruses^15^,^25^. Therefore, iNOS is an important signature molecule for M1 macrophage activation. Although increasing evidence indicates that iNOS is involved in the modulation of immune responses in addition to its killing effect of pathogens, the importance of iNOS involved in the control of M1 macrophage differentiation is incompletely understood. Previously we demonstrated that IL-12 mRNA and protein expression were significantly increased in iNOS KO mice, indicating that iNOS indeed contributes the regulation of proinflammatory cytokines by macrophages^18^. In addition, Giordano *et al*.^26^ reported that expression of inflammatory cytokines including TNF-α, IL-6, IL-12p70 and IL-23 was upregulated in *iNOS*^*−/−*^ bone marrow-derived dendritic cells. Taken together, these results indicate that iNOS expressed in innate immune cells including macrophages can modulate inflammatory cytokine production, and these cytokines are generally produced by M1 macrophages.

In the present study, we clearly demonstrated that iNOS expressed by M1 macrophages plays a negative role in the regulation of M1 macrophage differentiation. This conclusion was supported by the following observations: (1) the expression of M1 macrophage signature genes is significantly increased in *iNOS*^*−/−*^ M1 macrophages while eNOS and nNOS deficiency has no effect on M1 macrophage differentiation; (2) *iNOS*^*−/−*^ macrophages induce strong T-cell activation; (3). The iNOS-selective inhibitor, L-NIL, significantly increased the expression of M1 macrophage signature genes. In addition, the NO donor, SNAP, significantly suppressed M1 macrophage differentiation; (4) C57BL/6 mice transferred with *iNOS*^*−/−*^ macrophages are more susceptible to endotoxin shock with enhanced M1 macrophage cytokines. Thus, our observations support the concept that iNOS expressed in M1 macrophages modulates M1 macrophage differentiation by regulation M1 macrophage signature gene expressions.

IL-10 is an anti-inflammatory cytokine and has been reported to inhibit macrophage function, resulting in the control of inflammation. IL-10 can be produced by different cells types including macrophages, T cells and B cells. It is well established that M2 macrophages produce IL-10 resulting in the modulation of immune response. To exclude the possibility that the enhanced M1 macrophage differentiation was due to effects of IL-10 in *iNOS*^*−/−*^ mice, we examined IL-10 expression in WT and *iNOS*^*−/−*^ macrophages under M1 conditions. IL-10 mRNA expression was comparable in WT and *iNOS*^*−/−*^ macrophages. Furthermore, we found that NO donor SNAP significantly suppressed the expression of M1 macrophage signature gene expression. This indicated that enhanced M1 macrophage differentiation in *iNOS*^*−/−*^ mice is independent of the effects of IL-10.

IRF5, a member of interferon regulatory factor (IRF) family, has a variety of functions including activation of genes encoding inflammatory cytokines, type I interferon and tumour suppressors^3^,^27^,^28^,^29^. Recently because M1 macrophages expressed high amount of IRF5 and overexpression of IRF5 in M2 macrophages induced global expression of M1 macrophage signature genes, IRF5 has been defined as a key transcription factor for M1 macrophage differentiation^7^. In addition, IRF5-deficient mice are resistant to lethal endotoxin shock. The evidence suggests that activation of IRF5 expression defines commitment to the M1 macrophage lineage. Interestingly, microarray analysis showed that IRF5 expression in *iNOS*^*−/−*^ M1 macrophages was comparable to WT cells. Furthermore, SNAP or L-NIL had no significant effect on IRF5 protein expression in WT and *iNOS*^*−/−*^ M1 macrophages, indicating that the enhanced M1 macrophage differentiation is not the result of increased IRF5 protein levels. However, knockdown of IRF5 in *iNOS*^*−/−*^ M1 macrophages reduced the expression of M1 signature cytokines, suggesting that iNOS may control IRF5 activation during M1 macrophage differentiation. How might iNOS regulate IRF5 activation? Recently we have shown that tyrosine residues in RORγt are nitrated. Such nitration of RORγt significantly impaired the binding of RORγt to the promoter region of the *IL-17* gene, resulting in the inhibition of IL-17 transcription^30^. We assume that iNOS-derived NO may also nitrates tyrosine residues of IRF5 protein. The results indeed showed that the tyrosine residues in IRF5 protein were nitrated in WT M1 macrophages but not in *iNOS*^*−/−*^ M1 macrophages. In addition, ChIP assay showed that IRF5 DNA binding activity was clearly enhanced in *iNOS*^*−/−*^ macrophages and SNAP suppressed IRF5 DNA binding activity. Furthermore, we identified Tyr74 and Tyr104 residues of IRF5 are important for induction of IL-12p40 promoter activation. A previous study demonstrated that tyrosines of IκBα are nitrated as a consequence of NO synthase activation, resulting in dissociation of IκBα from NF-κB^31^. Some other studies have presented that nitration of a specific tyrosine in proteins can have structural and functional significance^32^,^33^,^34^. Taken together, our study reveals a novel mechanism for the modulation of M1 macrophage differentiation by nitration of IRF5 tyrosine residues.

M1 macrophages have been believed to play critical roles in host defense against bacterial infection, endotoxin shock and tumour growth^9^,^10^,^35^,^36^,^37^. It is well known that iNOS-derived NO contributes to the direct killing of various pathogens. However, the modulation of iNOS on M1 macrophage differentiation *in vivo* is still unknown. In the present study, we found that iNOS-deficient mice were susceptible to *L.monoctygenes* infection as reported previously since NO is directly involved in the killing of bacteria. However, the expression of M1 macrophage signature molecules was significantly increased in iNOS-deficient mice after infection, indicating that M1 macrophage activation was enhanced in iNOS-deficient mice during *L.monoctygenes* infection. Consistent with the results, mice transferred with *iNOS*^*−/−*^ macrophages were susceptible to lethal endotoxin shock, with enhanced expression of M1 macrophage signature molecules. Thus, these results suggest that iNOS expressed in macrophages plays a negative role in M1 macrophage differentiation *in vivo*, although it is a key marker for M1 macrophages. In another *in vivo* model of tumour inoculation of melanomas, L-NIL treatment significantly decreased the tumour size in tumour-bearing mice. In addition, tumour-infiltrating cell analysis showed that L-NIL treatment significantly enhanced the percentage of M1 macrophages in the tumour microenvironment. Thus, iNOS inhibition in tumour microenvironment facilitates M1 macrophage differentiation, resulting in the decrease of tumour size, further confirming that NO negatively regulates M1 macrophage differentiation *in vivo*.

Our systems analyses reveal a scenario that may best reflect and explain dynamic and balanced innate immune modulation in real-life settings. *In vivo*, macrophages likely exist as a mixed population of M1/M2 or hybrid phenotypes instead of an exclusively polarized M1 or M2 state^38^. Balanced macrophage portfolio would enable proper adaptation to changing environments. Similar phenomena are increasingly recognized in other cellular systems such as T-helper cells^39^. Our recent computational studies reconciled the current debate regarding the ‘plasticity’ versus ‘stability’ of T-helper cells^40^. A dynamic and mutually inhibitory motif differentially senses the signal strength activating the couple induction of Th17 and Treg cells^40^. On the basis of external signal strength, CD4 T-helper cells may readily adopt Th17, Treg or a double-positive phenotypes^40^. This would allow timely immune activation and homeostasis. Similarly, our current study provides initial evidence that macrophages possess similar dynamic circuit capable of fine-tuning the expression of iNOS and IL-12.

Taken together, our studies clearly demonstrate that in addition to acting as a signature marker for M1 macrophage cells, iNOS expressed in M1 macrophages dedifferentiate M1 macrophages. According to our observations, we suggest a novel molecular mechanism for the effects of iNOS-derived NO on M1 macrophage dedifferentiation that involves the suppression of IRF5 activation. The results established a novel concept that iNOS expressed in macrophages not only selectively regulates M1 macrophage gene expression but also modulates the M1 macrophage dedifferentiation, as a whole resulting in control of innate immune responses.

## Methods

### Mice

C57BL/6J(B6, stock#000664), iNOS-deficient mice (B6.129P2-*Nos2*^*tm1Lau*^/J, stock#002609), eNOS-deficient mice (B6.129P2-*Nos3*^*tm1unc*^/J, stock#002684), nNOS^*−/−*^ mice (B6.129S4-*Nos1*^*tm2pih*^/J, stock#008519) and CD4^+^ OVA TCR transgenic (OT-II) mice (B6. Cg-Tg (TcraTcrb)425Cbn/J, stock#004194) were obtained from Jackson laboratory and maintained in the barrier facility at the Mount Sinai School of Medicine. For all the experiments, 6- to 8-week-old female mice were used. The animal study protocols were approved by the Institutional Animal Care and Use Committees of Mount Sinai School of Medicine and Virginia Tech.

### Antibodies

The following antibodies were used at the indicated dilutions for immunoblotting and immunoprecipitation. Antibodies for Ub (sc-8017, 1:300), IRF1 (sc-640, 1:300), IRF4 (sc-6059, 1:300), IRF7 (sc-9083, 1:300), IRF8 (sc-6058, 1:300) and Nitrotyrosine (sc-65385, WB 1:300, IP 2 μg per 500 μg of total protein) were from Santa Cruz Biotechnology (USA). Antibodies for STAT1 (7649, 1:1,000) and phosho-STAT1 (9172, 1:1,000) were purchased from Cell Signaling Technology (USA). Antibodies for IRF5 (ab-21689, 1:1,000) were from Abcam (USA). For Chromatin immunoprecipitation, the following antibodies were applied: phosho-STAT1 (9172, 1:50), from Cell Signaling Technology (USA); IRF5 (ab-21689, 2 μg ml^−1^), obtained from Abcam (USA). For flow cytometry, fluorescently labelled antibodies to CD11b (M1/70, fluorescein isothiocyanate-labelled), MHC II (M5/114.15.2, alexa flour 700-labelled), Gr-1(RB6-8C5, fluorescein isothiocyanate-labelled), NOS2 (CXNFT, APC-labelled), IFN-γ (XMG1.2, APC-labelled, CD4 (GK1.5, fluorescein isothiocyanate-labelled), CD44 (IM7, APC-labelled) were all from eBioscience (USA), and were used at a 1:100 dilution. Antibodies for IL12 P40 (C15.6, PE-labelled) and CD25 (#558642, PE-labelled) were from BD Bioscience at a dilution of 1:100. Antibody for IL-1R (JAMA-147, APC-labelled) from Biolegend was diluted at 1:100.

### Preparation of bone marrow-derived macrophages

Bone marrow (BM) cells were isolated from tibias and femurs of C57BL/6 mice, and the cells were cultured in the complete DMEM medium supplemented with GM-CSF (10 ng ml^−1^) or M-CSF (10 ng ml^−1^). On day 6 or 7, bone marrow-derived macrophages (BMDMs) were harvested and then seeded in fresh complete DMEM medium at a density of 2 × 10^6^ cells ml^−1^ for experiments.

### Intracellular staining and flow cytometry

Bone marrow-derived macrophages were either activated with LPS (200 ng ml^−1^) plus IFN-γ (10 ng ml^−1^) or LPS alone at different concentrations overnight, and brefeldin A was added to the culture for 5 h before intracellular staining. Cells were fixed with IC Fixation Buffer (BD Bioscience), incubated with permeabilization buffer and stained with PE-anti-mouse IL-12 p40, APC-anti-iNOS and PE-Cy 5.5 anti-mouse C11b antibodies. Flow Cytometry was performed on a FACSCalibur (BD Biosciences).

### RNA isolation and quantitative real-time RT–PCR (qPCR)

Total RNA was extracted using an RNeasy plus kit (QIAGEN, Valencia, CA), and cDNA was generated with an oligo (dT) primer and the Superscript II system (Invitrogen, USA) followed by analysis using iCycler PCR with SYBR Green PCR master Mix (Applied Biosystems). Program was chosen to compare the CT value of target gene to housekeeping gene (ubiquitin) in a single sample, using the formula: 10000x2^ΔΔCT^. The following primer sets were used: IL-6 sense, 5′- CCAGAAACCGCTATGAAGTTCCT -3′, IL-6 anti-sense, 5′- CACCAGCATCAGTCCCAAG -A-3′; IL-12 P40 anti-sense, 5′- TCTTCAAAGGCTTCATCTGCAA -3′; TNFα sense, 5′- GCC-ACCACGCTCTTCTGTCT -3′, TNFα anti-sense, 5′- GGTCTGGGCCATAGAACTGATG -3′; ubiquitin sense, 5′- TGGCTATTAATTATTCGGTCTGCAT -3′, ubiquitin anti-sense, 5′- GCA- AGTGGCTAGAGTGCAGAGTAA -3′; iNOS sense, 5′- CCGAAGCAAACATCACATTCA -3′, iNOS anti-sense, 5′- GGTCTAAAGGCTCCGGGCT -3′; IRF5 sense, 5′- AATACCCCACC-ACCTTTTGA -3′, IRF5 anti-sense, 5′- TTGAGATCCGGGTTTGAGAT -3′; 5′- TGG-CTATTAATTATTCGGTCTGCA -3′; IRF4 sense, 5′- AGTCCCTTATTCTTTCACTTCA-TTTCCTTCC -3′, IRF4 anti-sense, 5′- GGAAGGAAATGAAGTGAAAGAATAAGGGACT -3′; Arg sense, 5′- CTCCAAGCCAAAGTCCTTAGAG -3′, Arg anti-sense, 5′- AGGAGCTGTCA-TTAGGGACATC -3′; CXCL10 (IP-10) sense, 5′- GACGGTCCGCTGCAACTG -3′, CXCL10 (IP-10) anti-sense, 5′- GCTTCCCTATGGCCCTCATT -3′; CXCL9 (MIG) sense, 5′- TGCACGATGCTCCTGCA -3′, CXCL9 (MIG) anti-sense, 5′- AGGTCTTTGAGGGATT-TGTAGTGG -3′; Ubiquitin sense, 5′- TGGCTATTAATTATTCGGTCTGCAT -3′, Ubiquitin antisense, GCAAGTGGCTAGAGTGCAGAGTAA -3′.

*Microarray analysis*. RNA microarray was performed using RNA isolated from WT and iNOS-deficient macrophages stimulated with LPS/IFNγ for 6 h. Total RNA was extracted using a RNeasy plus kit (QIAGEN, Valencia, CA), and the array was performed on an Illumina MouseWG-8 v2.0 expression beadchip (Illumina, USA) by the Genomics Core Facility at the Mount Sinai School of Medicine. Microarray data were normalized with background subtraction and rank invariant normalization. The value ‘1’ substituted any negative values for calculating fold change. All signals were Log2 transformed. Linear modelling of the transformed data was determined by using Limma in R with the Benjamini and Hochberg correction. Probe sets were selected based on fold change and *P* values. The expression values were plotted with heatmap.2. Accession number is available from the NCBI under GEO accession number (GSE65436).

### Transfection and luciferase reporter assay

The 293T cells were transiently transfected with an IL-12 p40 promoter luciferase reporter plasmid together with IRF5 in the presence of SNAP at different concentrations. For each transfection, 2.0 μg of plasmid was mixed with 100 μl of DMEM (without serum and antibiotics) and 4.0 μl of Lipofectamine 2000 reagent. The mixture was incubated at room temperature for 20 min and added to 12-well plates containing cells and complete medium. The cells were incubated for 30 h and harvested using reporter lysis buffer (Promega) for determination of luciferase activity. Cells were co-transfected with a β-galactosidase reporter plasmid to normalize experiments for transfection efficiency.

### T-cell proliferation assay

CD4^+^ T cells were purified from spleens and lymph nodes of OTII mice and the cells were labelled with CFSE. The labelled cells (1 × 10^5^ per well) were co-cultured with macrophages in the absence or presence of OVA_323-339_ peptide for 3 days in 96-well microplates. CFSE dilution assay was performed using flowcytometry.

### Immunoblotting analysis

Cells were washed with cold phosphate-buffered saline and lysed for 15 min on ice in 0.5 ml of lysis buffer (50 mM Tris-HCl, pH 8.0, 280 mM NaCl, 0.5% Nonidet P-40, 0.2 mM EDTA, 2 mM EGTA, 10% glycerol and 1 mM dithiothreitol) containing protease inhibitors. Cell lysates were clarified by centrifugation (4 °C, 15 min, 14,000 r.p.m.) and protein was subjected to 10% sodium dodecyl sulfate–PAGE (SDS–PAGE) and immunoblotting was performed. Anti-iNOS (Santa Cruz), anti-IRF5 (MBL), anti-STAT1 and anti-β-actin (Sigma) antibodies were used according to the manufactures’ instructions. Secondary antibodies were from Santa Cruz. The full scans of western blots are provided in the Supplementary Figs 13–18.

### Adoptive transfer and endotoxin-induced model of sepsis

C57BL/6 received 2 × 10^6^ WT or *iNOS*^*−/−*^ peritoneal macrophages or BMDMs. Mice were rested for 24 h and sepsis was induced by injecting 800 μg *E.coli*-derived ultra-pure LPS per mouse or PBS i.p. Survival after LPS was monitored and mice were killed immediately at a humane end point after noted by loss of self-righting (capability to right itself after falling) and insensitivity to touch. For serum collection, mice were injected i.p. with LPS (800 μg per mouse) and sera were collected 6 h later. Spleens were collected as well.

### Chromatin immunoprecipitation assay

The ChIP procedure was performed using an assay kit following the manufacturer’s instruction (Upstate Biotechnology, Lake Placid NY). In brief, activated macrophages were crosslinked by 1% formaldehyde for 10 min at 37 °C. Nuclei were prepared and subjected to sonication to obtain DNA fragments. Chromatin fractions were precleared with protein A-agarose beads followed by immunoprecipitation overnight at 4 °C with 3 μg of anti-IRF5 (Santa Cruz) or control antibody. Crosslinking was reversed at 65 °C for 4 h, followed by proteinase K digestion. DNA was purified and subjected to qPCR. The input DNA was diluted 200 times before PCR amplification. The input and immunoprecipitated DNA were amplified by qPCR using primers encompassing the IRF5-binding sites of the mouse IL-12 promoter regions.

### Infection of *L.monocytogenes* in mice

Mice were infected i.v. with 2 × 10^4^ CFU viable *L. monocytogenes* in a volume of 0. 2 ml PBS and mice were killed 2 days later^41^,^42^. Spleens and livers were homogenized and viable bacteria were grown on BHI agar plates and enumerated by the pour plate method after serial dilution. Colonies were enumerated 24 h after incubation at 37 °C. In addition, spleens and livers were homogenized in Trizol (Life Technologies, USA) and total RNA was extracted for q-PCR analysis. Sera were collected for cytokine determination.

### Tumour-bearing mice

For established tumour model, C57Bl/6 mice were injected s.c. with 3 × 10^5^ MT-RET-1 (mouse melanoma tumor cell line) cells in suspension. The MT-RET-1 tumour line^43^ is a melanoma line syngeneic with C57BL/6, which was developed by the laboratory of Willem Overwijk, UT MD Anderson Cancer Center, from a spontaneous melanoma occurring in a MT-RET transgenic mouse^44^. Mice were manually restrained, and the tumours were measured twice per week with calipers. Tumour sizes were determined according to the bidimensional product of the longest measurement multiplied by perpendicular. For characterization of the tumour microenvironment by flow cytometry (late tumour model), once tumours became established (30 mm^2^, around 2 weeks), half of the mice received L-NIL (2%) in drinking water for 7 days, and the other half received plain drinking water. After completing the course of L-NIL, all mice were killed and tumours were collected and processed into single-cell suspension. In brief, tumours were mashed on a filter mesh cup (Fisher). Ten milliliters RPMI medium 1640 (life technologies) with 1% FBS was added on mesh cup, and cells were centrifuged at 1,000 r.p.m. for 15 min at 4 °C. The pellet was resuspended in 5 ml ACK lysing buffer (Life Technologies) and incubated at room temperature for 5 min to remove red cells. Cells were washed with RPMI 1640 containing 1% FBS, and the pellet was resuspended in 1 ml complete RPMI medium. Tumour-infiltrating cells were isolated from the tumour using ficoll gradient and surface stained for CD11b and F4/80 markers and intracellularly stained for iNOS and arginase before analysis on the BD Fortessa. For tumour growth curves (early tumour model), mice were injected s.c. with 3 × 10^5^ MT-RET cells in suspension. Once tumours became palpable (4 mm^2^, around 4-5 days), half of the mice received L-NIL (2%) in drinking water for 18 days and the other half received plain drinking water.

### Model implementation

The basic motif shown in Fig. 7c was based on existing reports that suggest the existence of a mutually inhibitory circuit responsible for the generation of iNOS and IL-12 by varying dosages of LPS^22^,^23^,^24^,^45^,^46^. This motif was translated into ordinary differential equations (ODEs) to enable simulation. The framework we used to create the ODEs was not based on standard reaction rate equations, but rather on a formalism that allows us to capture complex dependencies in a simple manner^46^,^47^,^48^. We purposely developed this generic modelling approach that does not depend on minute details of complex pathways. Instead, this approach aimed at performing bifurcation analyses of integrated skeletal motifs to simulate potential cellular outcomes. We were able to derive dynamic properties representing the relative levels of iNOS and IL-12 expression in macrophages responding to varying TLR4 signal strength. Furthermore, stochastic terms were added to the ODEs to take into account random effects of intrinsic and extrinsic sources of noise^49^,^50^,^51^,^52^,^53^. In these equations, the iNOS and IL12 levels on a logarithm scale (with a base of 10) are represented by italicized variables *INOS* and *IL*12. We assume that both iNOS and IL12 levels have a dynamical range of 10-fold, so *INOS* and *IL12* vary between 0 and 1. The parameter *γ*_*i*_ determines the rates at which specie *i* (*i*=*INOS* or *IL12*) approach its time-varying ‘target’ value, 
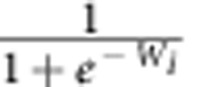
, which is a sigmoidal function varying between 0 and 1, with a value of ½ when *W*_*i*_=0. *W*_*i*_ is the net activation or inhibition on *i*, and its leading component, *ω*_*i*_, determines whether *i* is activated or inhibited when there are no regulatory signals impinging on *i* from any species in the motif. *ω*_*ij*_ is the strength of the influence from specie *j* to *i* (*ω*_*ij*_>0 for activation and<0 for inhibition). *S*_*i,j*_ represents the activity of intermediate signalling components, such as transcription factors, that are activated by *j* and control *i*'s expression (*i, j*=*INOS* or *IL12*). By applying quasi-equilibrium approximation, we use a sigmoidal function 
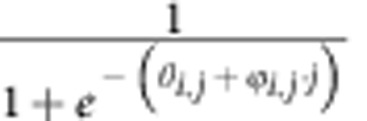
 to describe how *S*_*i,j*_ is controlled by *j*. Here the offset parameter *θ*_*i,j*_ determines whether *S*_*i,j*_ is activated or inhibited when there is no *j*. *ϕ*_*i,j*_ is the strength of the influence from specie *j* to *S*_*i,j.*_(*ϕ*_*ij*_>0 for activation and<0 for inhibition).


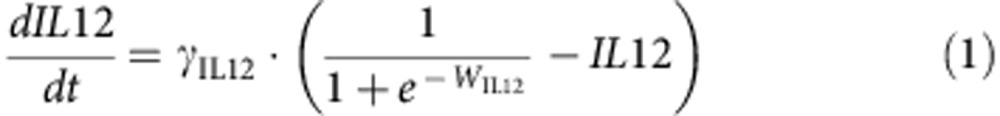



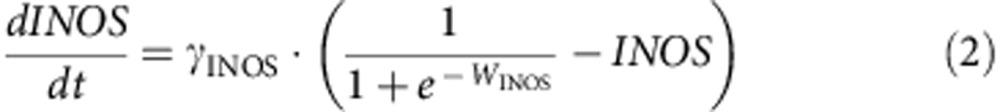



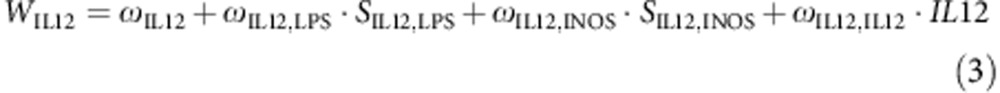



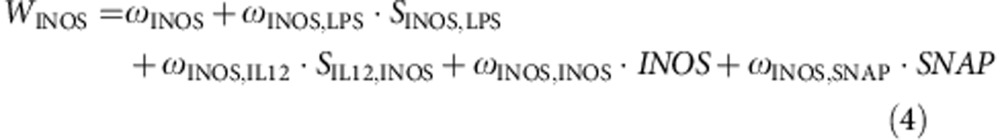



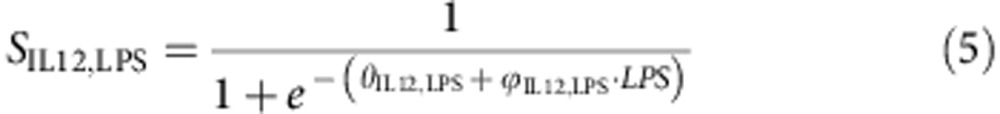



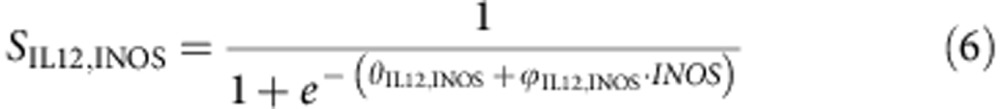



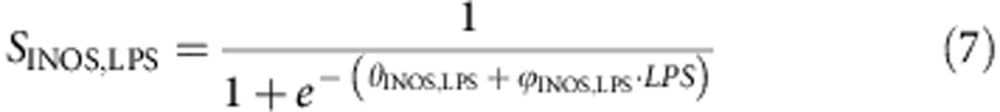



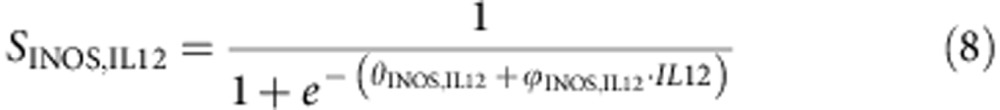


Please refer to Supplementary table 1 and Supplementary table 2 for the variables and parameter values. As we are modelling the motif in Fig. 7c at a high level of abstraction, none of the parameters in Supplementary Table 2 are directly related to measurable physical constants of the system. Rather, they are manually fitted to match the experimental observations on IL12 level at different conditions (*LPS*=0, 0.1, 1, 10 and 1,000 ng ml^−1^). Initially, the parameters for the deterministic part of the model were chosen to ensure that the system has at least two attractors (corresponding to IL12+ and IL12− states) separated by an unstable saddle. While LPS increases from 0 to 10 ng ml^−1^, the unstable saddle should move closer to the IL12− attractor. However, further increases of LPS should move the unstable saddle towards the IL12+ attractor. For the stochastic simulations (see method below), we chose appropriate noise parameter values to ensure that the system’s behaviour occurred in a manner similar to experimental observations. Please note that the noise and deterministic parameters are not independent. Thus, ultimately all the parameters were tuned in concert to qualitatively match the experimental results. We used Matlab (Version 7.9.0) to build the model and perform simulations.

### Stochastic simulation

Similar to refs 49, 51, noise terms were added to the differential equations for *IL12* and *INOS* to account for stochastic effects in the model, while the algebraic equations were left unchanged. The Langevin equation for variables *X* (*X*=*IL12* or *INOS*) follows the form:


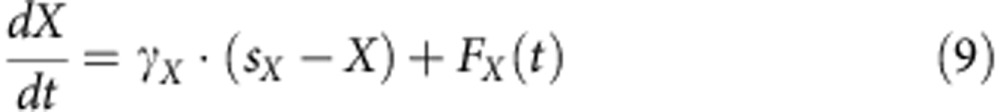


where *s*_X_ defines the steady-state level of *X* and *F*_X_(*t*) is a Gaussian white noise process. The equilibrium second moment of the variable *X*, <(*s*_X_−*X*)^2^*>*_eq_=*θ*_*X*_, is related to *γ*_X,_and the second moment of the noise by a fluctuation-dissipation theorem^52^,^53^ follows:


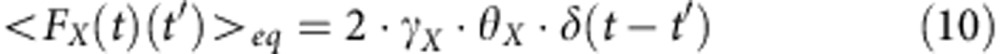


Thus, we can rewrite Equation 9 as:


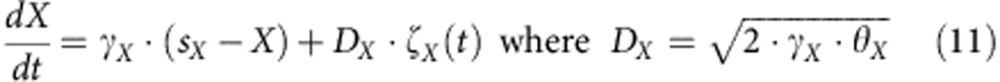


Here *ζ*_X_(*t*) is a temporally uncorrelated, statistically independent, Gaussian white noise process, which is formally defined by *ζ*_X_(*t*)≡lim_*dt*→0_ *N*(0,1/*dt*) with <*ζ*_X_(*t*)*ζ*_*X*_(*t*′)>=*δ*(*t*–*t*′). We set *γ*_X_=1 for all species for simplicity in our model. Thus, we can vary *D*_x_ as parameters to control the strength of noise to manually fit the model. Values of *D*_x_ are listed in Supplementary table 2.

The Langevin equations are integrated and propagated by the explicit method:


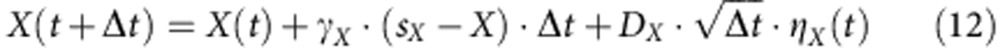


where the *η*_*i*_(*t*) are independent normal random variables. The stochastic simulations were performed in Matlab Version 7.9.0.

### Statistical analysis

The results are shown as means±s.e.m., and statistical analysis was performed using Student’s *t*-test. Where more than two groups were compared, one-way ANOVA with a Bonferroni's correction was performed; Kaplan–Meier method was used to estimate overall survival and the Log-rank test was applied to determine the difference of survival rate. *P* values <0.05 were considered statistically significant.

## Author contributions

G.L. and H.X. initiated the project. H.X. supervised the project. G.L., R.Z. and L.P. did most of the experiments. P.J., F.X., J.Y., Q.L., H.Z., K.S., J.W., X.L., Z.Z. and A.S. performed experiments. W.W., C.Q., C.S., J.H. and S.L. provided critical reagents and gave important advice throughout the study. S.G., C.C. and L.L. contributed to dynamic experimental analysis and computational modelling. G.L., L.L. and H.X. wrote the manuscript.

## Additional information

**How to cite this article:** Lu, G. *et al*. Myeloid cell-derived inducible nitric oxide synthase suppresses M1 macrophage polarization. *Nat. Commun.* 6:6676 doi: 10.1038/ncomms7676 (2015).

## Supplementary Material

Supplementary Information Supplementary Figures 1-18 and Supplementary Tables 1-2

## References

[b1] Biswas S. K. & Mantovani A. Macrophage plasticity and interaction with lymphocyte subsets: cancer as a paradigm. Nat. Immunol. 11, 889–896 (2010) . 20856220 10.1038/ni.1937

[b2] Martinez F. O., Helming L. & Gordon S. Alternative activation of macrophages: an immunologic functional perspective. Annu. Rev. Immunol. 27, 451–483 (2009) . 19105661 10.1146/annurev.immunol.021908.132532

[b3] Lawrence T. & Natoli G. Transcriptional regulation of macrophage polarization: enabling diversity with identity. Nat. Rev. Immunol. 11, 750–761 (2011) . 22025054 10.1038/nri3088

[b4] Ruffell B., Affara N. I. & Coussens L. M. Differential macrophage programming in the tumor microenvironment. Trends Immunol. 33, 119–126 (2012) . 22277903 10.1016/j.it.2011.12.001 PMC3294003

[b5] Sica A. & Mantovani A. Macrophage plasticity and polarization: in vivo veritas. J. Clin. Invest. 122, 787–795 (2012) . 22378047 10.1172/JCI59643 PMC3287223

[b6] Gordon S. & Martinez F. O. Alternative activation of macrophages: mechanism and functions. Immunity 32, 593–604 (2010) . 20510870 10.1016/j.immuni.2010.05.007

[b7] Krausgruber T. . IRF5 promotes inflammatory macrophage polarization and TH1-TH17 responses. Nat. Immunol. 12, 231–238 (2011) . 21240265 10.1038/ni.1990

[b8] Satoh T. . The Jmjd3-Irf4 axis regulates M2 macrophage polarization and host responses against helminth infection. Nat. Immunol. 11, 936–944 (2010) . 20729857 10.1038/ni.1920

[b9] Hristodorov D., Mladenov R., Huhn M., Barth S. & Thepen T. Macrophage-targeted therapy: CD64-based immunotoxins for treatment of chronic inflammatory diseases. Toxins 4, 676–694 (2012) . 23105975 10.3390/toxins4090676 PMC3475223

[b10] Cohen H. B. & Mosser D. M. Extrinsic and intrinsic control of macrophage inflammatory responses. J. Leukoc. Biol. 94, 913–919 (2013) . 23964115 10.1189/jlb.0413236 PMC4051261

[b11] Bogdan C. Nitric oxide and the immune response. Nat. Immunol. 2, 907–916 (2001) . 11577346 10.1038/ni1001-907

[b12] Calabrese V. . Nitric oxide in the central nervous system: neuroprotection versus neurotoxicity. Nat. Rev. Neurosci. 8, 766–775 (2007) . 17882254 10.1038/nrn2214

[b13] Griffith O. W. & Stuehr D. J. Nitric oxide synthases: properties and catalytic mechanism. Annu. Rev. Physiol. 57, 707–736 (1995) . 7539994 10.1146/annurev.ph.57.030195.003423

[b14] MacMicking J., Xie Q. W. & Nathan C. Nitric oxide and macrophage function. Annu. Rev. Immunol. 15, 323–350 (1997) . 9143691 10.1146/annurev.immunol.15.1.323

[b15] Bogdan C., Rollinghoff M. & Diefenbach A. The role of nitric oxide in innate immunity. Immunol. Rev. 173, 17–26 (2000) . 10719664 10.1034/j.1600-065x.2000.917307.x

[b16] Xiong H., Kawamura I., Nishibori T. & Mitsuyama M. Suppression of IFN-gamma production from Listeria monocytogenes-specific T cells by endogenously produced nitric oxide. Cell Immunol. 172, 118–125 (1996) . 8806814 10.1006/cimm.1996.0222

[b17] Niedbala W., Cai B. & Liew F. Y. Role of nitric oxide in the regulation of T cell functions. Ann. Rheum. Dis. 65, (Suppl 3): iii37–iii40 (2006) . 17038470 10.1136/ard.2006.058446 PMC1798386

[b18] Xiong H. . Inhibition of interleukin-12 p40 transcription and NF-kappaB activation by nitric oxide in murine macrophages and dendritic cells. J. Biol. Chem. 279, 10776–10783 (2004) . 14679201 10.1074/jbc.M313416200

[b19] Bogdan C. The multiplex function of nitric oxide in (auto)immunity. J. Exp. Med. 187, 1361–1365 (1998) . 9565628 10.1084/jem.187.9.1361 PMC2212276

[b20] Niedbala W. . Regulation of type 17 helper T-cell function by nitric oxide during inflammation. Proc. Natl Acad. Sci. USA 108, 9220–9225 (2011) . 21576463 10.1073/pnas.1100667108 PMC3107290

[b21] Maitra U. . Molecular mechanisms responsible for the selective and low-grade induction of proinflammatory mediators in murine macrophages by lipopolysaccharide. J. Immunol. 189, 1014–1023 (2012) . 22706082 10.4049/jimmunol.1200857 PMC3392521

[b22] Zhang X. & Morrison D. C. Lipopolysaccharide-induced selective priming effects on tumor necrosis factor alpha and nitric oxide production in mouse peritoneal macrophages. J. Exp. Med. 177, 511–516 (1993) . 8426119 10.1084/jem.177.2.511 PMC2190891

[b23] Morris M. C., Gilliam E. A., Button J. & Li L. Dynamic modulation of innate immune response by varying dosages of lipopolysaccharide (LPS) in human monocytic cells. J. Biol. Chem. 289, 21584–21590 (2014) . 24970893 10.1074/jbc.M114.583518 PMC4118118

[b24] Deng H., Maitra U., Morris M. & Li L. Molecular mechanism responsible for the priming of macrophage activation. J. Biol. Chem. 288, 3897–3906 (2013) . 23264622 10.1074/jbc.M112.424390 PMC3567643

[b25] Alderton W. K., Cooper C. E. & Knowles R. G. Nitric oxide synthases: structure, function and inhibition. Biochem. J. 357, 593–615 (2001) . 11463332 10.1042/0264-6021:3570593 PMC1221991

[b26] Giordano D. . Nitric oxide controls an inflammatory-like Ly6C(hi)PDCA1+ DC subset that regulates Th1 immune responses. J. Leukoc. Biol. 89, 443–455 (2011) . 21178115 10.1189/jlb.0610329 PMC3040463

[b27] Tamura T., Yanai H., Savitsky D. & Taniguchi T. The IRF family transcription factors in immunity and oncogenesis. Annu. Rev. Immunol. 26, 535–584 (2008) . 18303999 10.1146/annurev.immunol.26.021607.090400

[b28] Taniguchi T., Ogasawara K., Takaoka A. & Tanaka N. IRF family of transcription factors as regulators of host defense. Annu. Rev. Immunol. 19, 623–655 (2001) . 11244049 10.1146/annurev.immunol.19.1.623

[b29] Zhang R. . Regulation of pathogenic Th17 cell differentiation by IL-10 in the development of glomerulonephritis. Am. J. Pathol. 183, 402–412 (2013) . 23747510 10.1016/j.ajpath.2013.05.001 PMC3730759

[b30] Jianjun Y. . T cell-derived inducible nitric oxide synthase switches off Th17 cell differentiation. J. Exp. Med. 210, 1447–1462 (2013) . 23797094 10.1084/jem.20122494 PMC3698516

[b31] Yakovlev V. A. . Tyrosine nitration of IkappaBalpha: a novel mechanism for NF-kappaB activation. Biochemistry 46, 11671–11683 (2007) . 17910475 10.1021/bi701107z PMC2678910

[b32] Radi R. Nitric oxide, oxidants, and protein tyrosine nitration. Proc. Natl Acad. Sci. USA 101, 4003–4008 (2004) . 15020765 10.1073/pnas.0307446101 PMC384685

[b33] Ji Y., Neverova I., Van Eyk J. E. & Bennett B. M. Nitration of tyrosine 92 mediates the activation of rat microsomal glutathione s-transferase by peroxynitrite. J. Biol. Chem. 281, 1986–1991 (2006) . 16314419 10.1074/jbc.M509480200

[b34] Ischiropoulos H. & Gow A. Pathophysiological functions of nitric oxide-mediated protein modifications. Toxicology 208, 299–303 (2005) . 15691593 10.1016/j.tox.2004.11.018

[b35] Olefsky J. M. & Glass C. K. Macrophages, inflammation, and insulin resistance. Annu. Rev. Physiol. 72, 219–246 (2010) . 20148674 10.1146/annurev-physiol-021909-135846

[b36] Spence S. . Suppressors of cytokine signaling 2 and 3 diametrically control macrophage polarization. Immunity 38, 66–78 (2013) . 23177319 10.1016/j.immuni.2012.09.013

[b37] Mills C. D. M1 and M2 macrophages: oracles of health and disease. Crit. Rev. Immunol. 32, 463–488 (2012) . 23428224 10.1615/critrevimmunol.v32.i6.10

[b38] Mosser D. M. & Edwards J. P. Exploring the full spectrum of macrophage activation. Nat. Rev. Immunol. 8, 958–969 (2008) . 19029990 10.1038/nri2448 PMC2724991

[b39] Voo K. S. . Identification of IL-17-producing FOXP3+ regulatory T cells in humans. Proc. Natl Acad. Sci. USA 106, 4793–4798 (2009) . 19273860 10.1073/pnas.0900408106 PMC2653560

[b40] Hong T., Xing J., Li L. & Tyson J. J. A mathematical model for the reciprocal differentiation of T helper 17 cells and induced regulatory T cells. PLoS. Comput. Biol. 7, e1002122 (2011) . 21829337 10.1371/journal.pcbi.1002122 PMC3145653

[b41] Xiong H., Kawamura I., Nishibori T. & Mitsuyama M. Cytokine gene expression in mice at an early stage of infection with various strains of Listeria spp. differing in virulence. Infect. Immun. 62, 3649–3654 (1994) . 8063381 10.1128/iai.62.9.3649-3654.1994 PMC303014

[b42] Xiong H., Tanabe Y., Ohya S. & Mitsuyama M. Administration of killed bacteria together with listeriolysin O induces protective immunity against Listeria monocytogenes in mice. Immunology 94, 14–21 (1998) . 9708181 10.1046/j.1365-2567.1998.00477.x PMC1364325

[b43] Jayaraman P. . Tumor-expressed inducible nitric oxide synthase controls induction of functional myeloid-derived suppressor cells through modulation of vascular endothelial growth factor release. J. Immunol. 188, 5365–5376 (2012) . 22529296 10.4049/jimmunol.1103553 PMC3358566

[b44] Kato M. . Transgenic mouse model for skin malignant melanoma. Oncogene 17, 1885–1888 (1998) . 9778055 10.1038/sj.onc.1202077

[b45] Shnyra A., Brewington R., Alipio A., Amura C. & Morrison D. C. Reprogramming of lipopolysaccharide-primed macrophages is controlled by a counterbalanced production of IL-10 and IL-12. J. Immunol. 160, 3729–3736 (1998) . 9558074

[b46] Fu Y. . Network topologies and dynamics leading to endotoxin tolerance and priming in innate immune cells. PLoS. Comput. Biol. 8, e1002526 (2012) . 22615556 10.1371/journal.pcbi.1002526 PMC3355072

[b47] Mjolsness E., Sharp D. H. & Reinitz J. A connectionist model of development. J. Theor. Biol. 152, 429–453 (1991) . 1758194 10.1016/s0022-5193(05)80391-1

[b48] Wilson H. R. & Cowan J. D. Excitatory and inhibitory interactions in localized populations of model neurons. Biophys. J. 12, 1–24 (1972) . 4332108 10.1016/S0006-3495(72)86068-5 PMC1484078

[b49] Chen C., Baumann W. T., Clarke R. & Tyson J. J. Modeling the estrogen receptor to growth factor receptor signaling switch in human breast cancer cells. FEBS Lett. 587, 3327–3334 (2013) . 23994522 10.1016/j.febslet.2013.08.022 PMC3893882

[b50] Tyson J. J. & Novak B. Functional motifs in biochemical reaction networks. Annu. Rev. Phys. Chem. 61, 219–240 (2010) . 20055671 10.1146/annurev.physchem.012809.103457 PMC3773234

[b51] Tyson J. J. . Dynamic modelling of oestrogen signalling and cell fate in breast cancer cells. Nat. Rev. Cancer 11, 523–532 (2011) . 21677677 10.1038/nrc3081 PMC3294292

[b52] Kubo R. The fluctuation-dissipation theorem. Rep. Prog. Phys. 29, 30 (1966) .

[b53] Zwanzig R. Dynamic disorder—passage through a fluctuating bottleneck. J. Chem. Phys. 97, 3587–3589 (1992) .

