# Supplementary material for: Myeloid cell-derived inducible nitric oxide synthase suppresses M1 macrophage polarization
Source: Nat Commun. 2015 Mar 27;6:6676. doi: 10.1038/ncomms7676 (PMC4389243; doi:10.1038/ncomms7676)
Supplement: Supplementary Information — Supplementary Figures 1-18 and Supplementary Tables 1-2 [file ncomms7676-s1.pdf]

Supplementary Figure 1

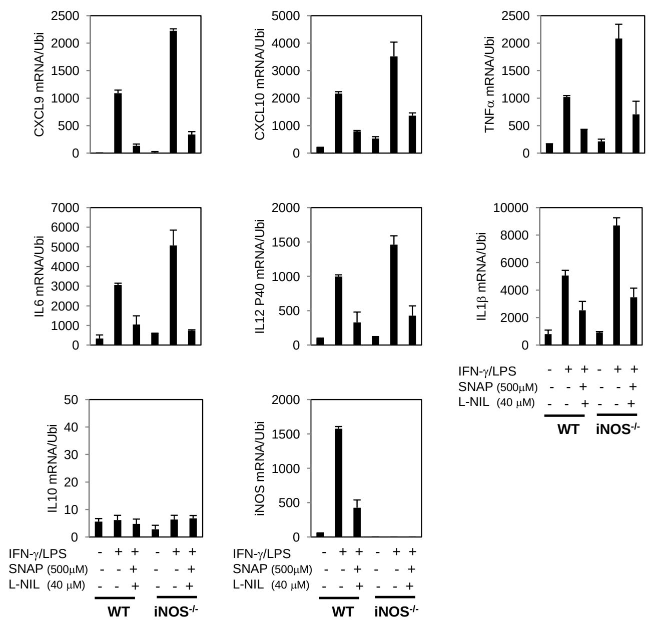

Supplementary Figure 1. Enhanced M1 macrophage signature gene expression in iNOS-deficient mice. Bone marrow cells were incubated with M-CSF (10 ng ml^{-1}) for one week. The cells were then stimulated with IFN-γ (10 ng ml^{-1}) plus LPS (200 ng ml^{-1}) for 6 hrs and mRNA expression of indicated genes was determined by qPCR. The data shown were normalized to levels of ubiquitin expression. The results were representative of two independent experiments.

Supplementary Figure 2

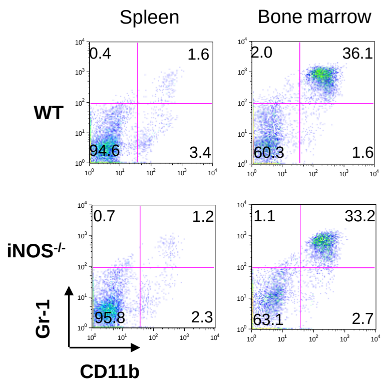

Supplementary Figure 2. Normal myeloid cell development in *iNOS*$^{-/-}$ mice. Spleen cells and bone marrow cells were prepared from WT and *iNOS*$^{-/-}$ mice and the cells were stained for Gr-1 and CD11b and analyzed by flow cytometry.

Supplementary Figure 3

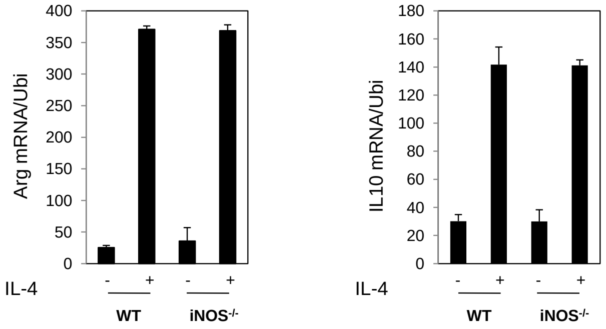

Supplementary Figure 3. iNOS defienecy has no effect on mRNA expression of Arginase and IL-10. BMDMs from WT or *iNOS*$^{-/-}$ mice were stimulated with IL-4 (10 ng ml^{-1}) for 6 hrs and the total cellular RNA was extracted. qPCR was performed for the analysis of mRNA expression of Arginase and IL-10. The results were representative of two independent experiments.

Supplementary Figure 4

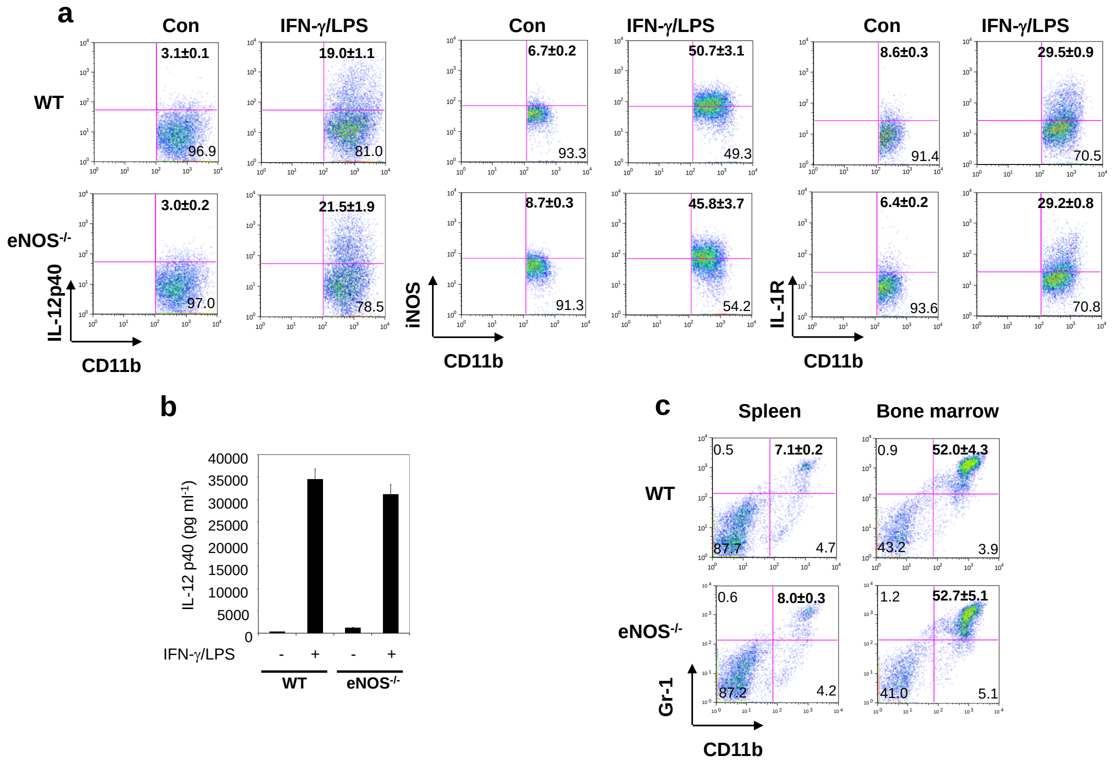

Supplementary Figure 4. *eNOS* deficiency has no significant effect on M1 macropahge differentiation. (**a**) BMDMs from wild type or *eNOS*$^{-/-}$ mice were stimulated with IFN-γ (10 ng ml^{-1}) plus LPS (200 ng ml^{-1}) for 24 hrs, stained for intracellular IL-12 p40, iNOS, and surface IL-1R and analyzed by flow cytometry. Representative FACS dot plots gated on CD11b^{+} cells and the percentages of IL-12-producing, iNOS expressing, and IL-1R positive CD11b^{+} cells are shown. (**b**) BMDMs from WT or *eNOS*$^{-/-}$ mice were stimulated with IFN-γ (10 ng ml^{-1}) plus LPS (200 ng ml^{-1}) for 24 hrs and supernatants were harvested for the analysis of IL-12 p40 production by ELISA. (**c**) Spleen cells and bone marrow cells were prepared from WT and *eNOS*$^{-/-}$ mice and the cells were stained for Gr-1 and CD11b and analyzed by flow cytometry. The results were representative of two independent experiments.

Supplementary Figure 5

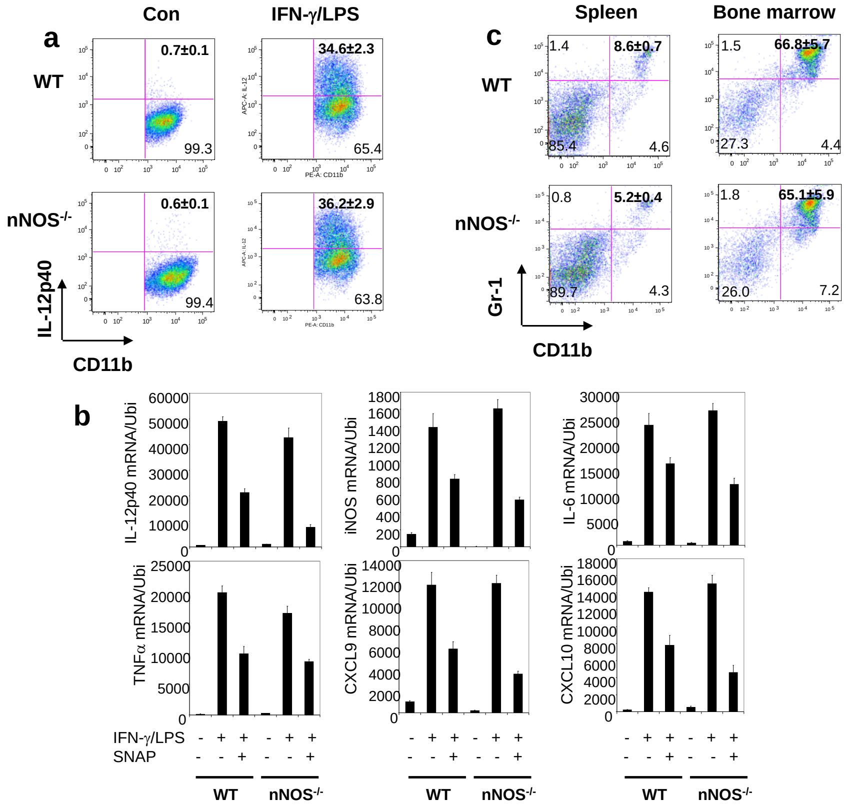

Supplementary Figure 5. *nNOS* deficiency has no significant effect on M1 macrophage differentiation. (**a**) BMDMs from wild type or *nNOS*$^{-/-}$ mice were stimulated with IFN-γ (10 ng ml^{-1}) plus LPS (200 ng ml^{-1}) for 24 hrs, stained for intracellular IL-12 p40 and analyzed by flow cytometry. Representative FACS dot plots gated on CD11b^{+} cells and the percentages of IL-12-producing CD11b^{+} cells are shown. (**b**) BMDMs from wild type or *nNOS*$^{-/-}$ mice were stimulated with IFN-γ (10 ng ml^{-1}) plus LPS (200 ng ml^{-1}) in the presence of SNAP (500 μM) for 6 hrs and total cellular RNA was extracted. qPCR was performed for the analysis of mRNA expression of indicated genes. (**c**) Spleen cells and bone marrow cells were prepared from WT and *nNOS*$^{-/-}$ mice and the cells were stained for Gr-1 and CD11b and analyzed by flow cytometry. The results were representative of two independent experiments.

Supplementary Figure 6

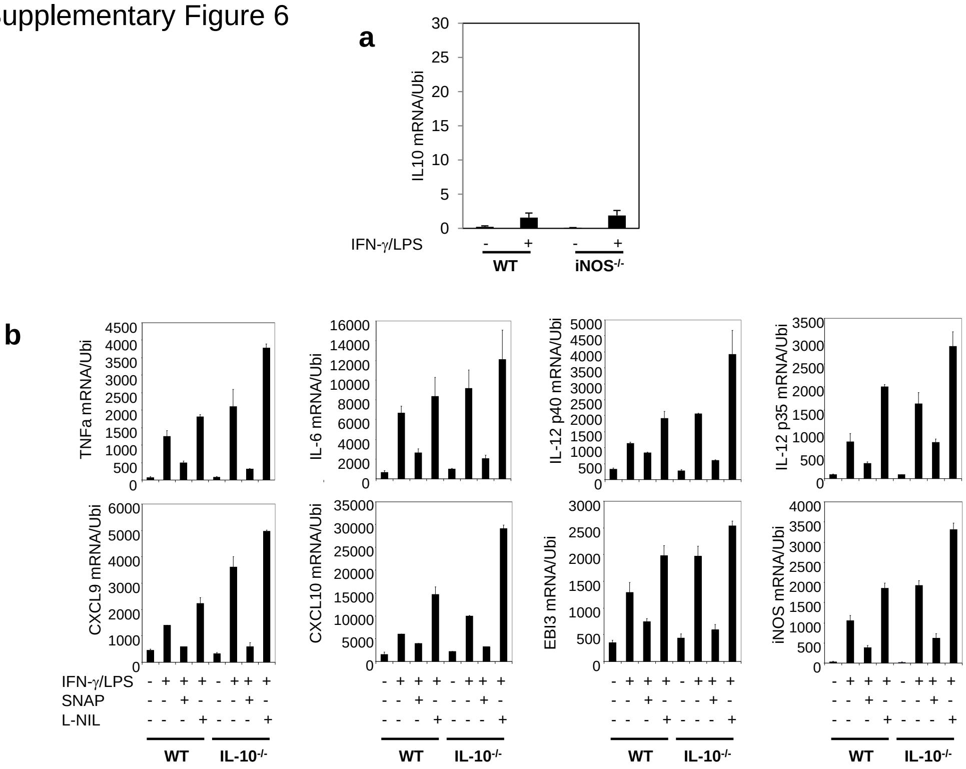

Supplementary Figure 6. iNOS suppresses M1 macrophage differentiation independent of IL-10. (**a**) BMDMs from wild type or *iNOS*$^{-/-}$ mice were stimulated with IFN-γ (10 ng ml^{-1}) plus LPS (200 ng ml^{-1}) for 6 hrs and total cellular RNA was extracted. qPCR was performed for the analysis of IL-10 mRNA expression. (**b**) BMDMs from wild type or *IL-10*$^{-/-}$ mice were stimulated with IFN-γ (10 ng ml^{-1}) plus LPS (200 ng ml^{-1}) in the presence of SNAP (500 μM) or IL-NIL (40 μM) for 6 hrs and total cellular RNA was extracted. qPCR was performed for the analysis of mRNA expression of indicated genes. The results are representative of three similar experiments.

Supplementary Figure 7

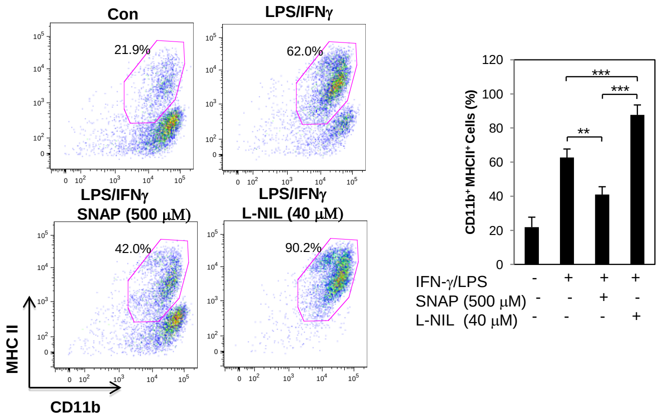

Supplementary Figure 7. Nitric oxide suppresses MHCII expression. BMDMs from WT mice were with IFN-γ (10 ng ml^{-1}) plus LPS (200 ng ml^{-1}) in the presence of SNAP (500 μM) or LNIL (40 μM) for 24 hrs, stained for surface MHC II and analyzed by flow cytometry. Representative FACS dot plots gated on $CD11b^+$ cells and the percentage of MHC II positive $CD11b^+$ cells are shown. Each bar represents mean ± SD from three independent experiments, one-way ANOVA with a Bonferroni correction, $**P<0.01$; $***P<0.001$.

Supplementary Figure 8

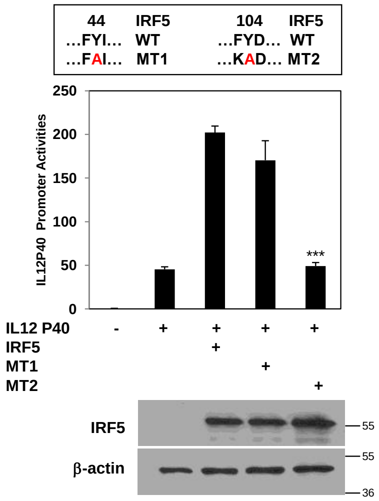

Supplementary Figure 8. IRF5 induces IL-12 p40 promoter activation. 293T cells were transfected with an IL-12 p40 promoter reporter construct and wild type IRF5 or mutant IRF5Y44F or mutant IRF5Y104F plasmids for 30 hrs. Luciferase assays were performed and luciferase activities were normalized to β-galactosidase activity. In addition, IRF5 protein expression was analyzed by western blotting. Data indicate mean ± SD of triplicate cultures and are representative of three independent experiments, unpaired Student's *t*-test, ***P<0.001 versus cells transfected with WT IRF5 plasmid.

Supplementary Figure 9

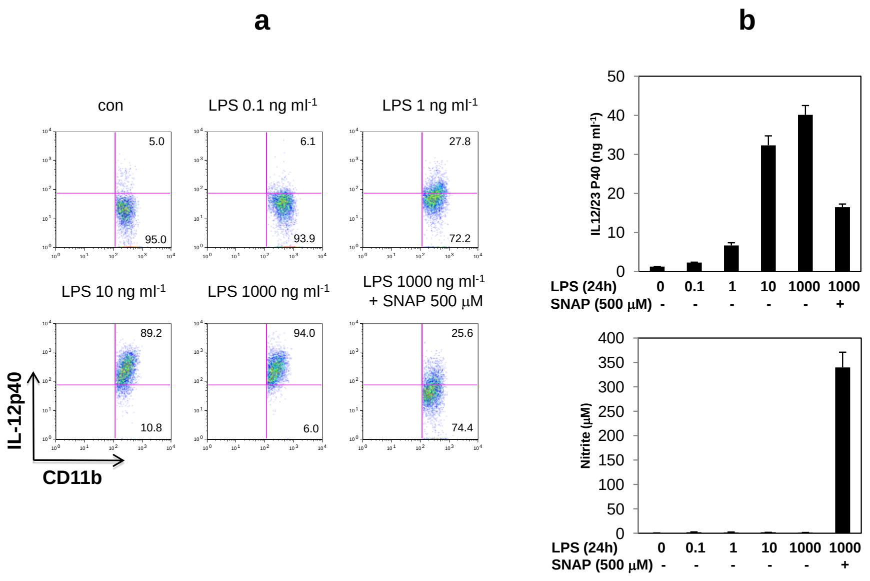

Supplementary Figure 9. IL-12 production in iNOS deficient macrophages. (**a**) BMDMs from *iNOS*$^{-/-}$ mice were treated with different doses of LPS and SNAP overnight, and stained for intracellular IL-12 and surface CD11b^{+} and analyzed by flow cytometry. Representative FACS dot plots gated on CD11b^{+} cells and the percentages of IL-12-producing cells are shown. **(b)** The supernatants were analyzed for IL-12 and nitrite production. Each bar represents mean ± SD from three independent experiments.

Supplementary Figure 10

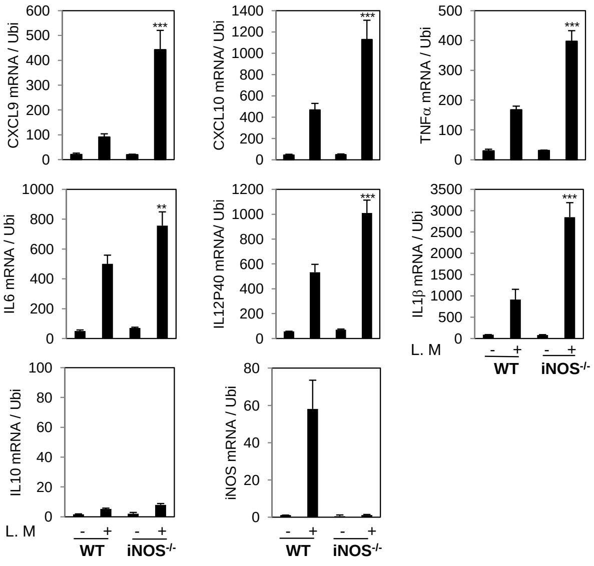

Supplementary Figure 10. iNOS deficiency promotes the M1 macrophage differentiation *in vivo*. WT or *iNOS*$^{-/-}$ mice were injected (i.p.) with 1 x 10^5 CFU of viable *L. monocytogenes* for 12 hrs. The peritoneal macrophages were harvested and qPCR was performed for the analysis of M1 macrophages signature gene mRNA expression. Each bar represents mean ± SD from three independent experiments, unpaired Student's *t*-test, **P<0.01, ***P<0.001 versus WT injected with *L. monocytogenes*.

Supplementary Figure 11

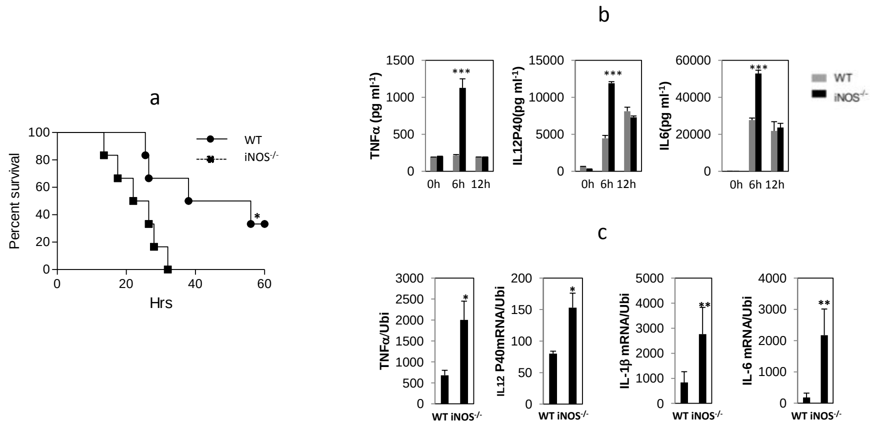

Supplementary Figure 11. iNOS deficiency promotes the M1 macrophage differentiation *in vivo*. (a) C57Bl/6 mice were transferred with 2 x 10^6 WT or *iNOS*$^{-/-}$ BMDMs for 24 hours and the recipient mice were then challenged with LPS (800 μg per mouse). The survival of mice was observed (n=8 in each group). Kaplan-Meier method was used to estimate overall survival and the Log-rank test was applied to determine the difference of survival rate. *P<0.05. (**b**) The recipient mice were challenged with LPS (800 μg per mouse) for 6 hrs and 12 hrs and sera level of cytokines was determined by ELISA. Each bar represents mean ± SD from three independent experiments, unpaired Student's *t*-test, ***P<0.001. (**c**) The recipient mice were challenged with LPS (800 μg per mouse) for 6 hrs and spleens were removed. Total cellular RNA was extracted and qPCR was performed for the analysis of mRNA expression of indicated genes. Each bar represents mean ± SD from three independent experiments, unpaired Student's *t*-test, *P< 0.05; **P<0.01.

Supplementary Figure 12

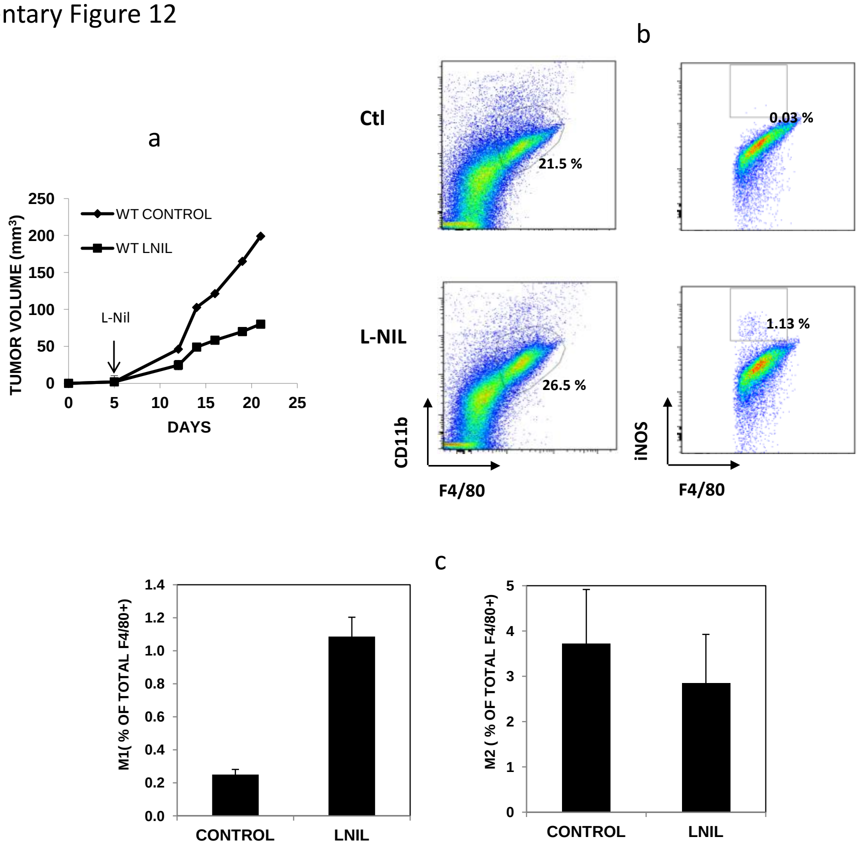

Supplementary Figure 12. Pharmacologic iNOS inhibition decreases tumor growth and increases M1 macrophage accumulation. (a) Growth of untreated and early (day 4) L-NIL treated MT-RET-1 melanoma in C57BL/6 mice. **(b)** Representative FACS plots of percentages of tumor infiltrating total ($CD11b^+F4/80^+$ cells) and M1 ($iNOS^+$) and M2 ($arginase^+$) macrophages. (**c**) M1 and M2 macrophage populations in the tumor expressed as a percentage of total $CD11b^+F4/80^+$ macrophages in control and L-NIL-treated MT-RET-bearing mice on day 21 after tumor injection. L-NIL treatment significantly increases the population of intratumoral M1 macrophages compared to the control group, without significantly altering M2 macrophage levels. Each graph shown represents pooled data from at least three experiments with n=5 mice per group.

Supplementary Figure 13

Fig. 3a

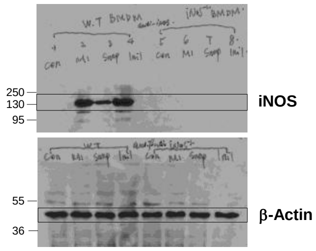

Fig. 3b

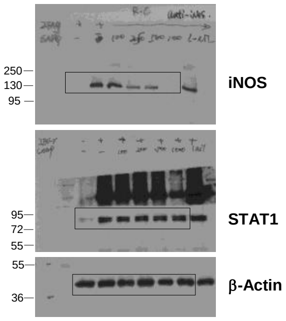

Fig. 3f

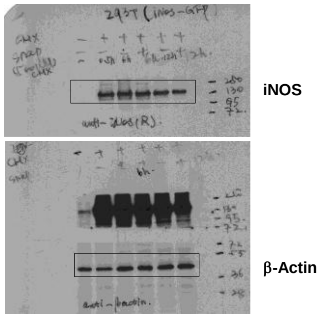

Supplementary Figure 13: full scans of blots in Figure3a, Figure 3b and Figure 3f

Supplementary Figure 14

Fig. 3g

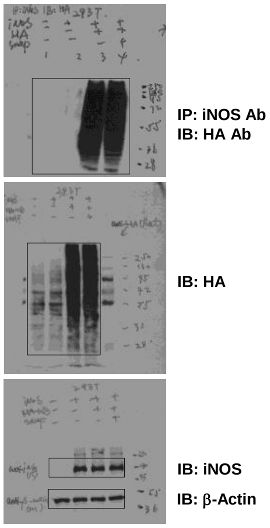

Fig. 3h

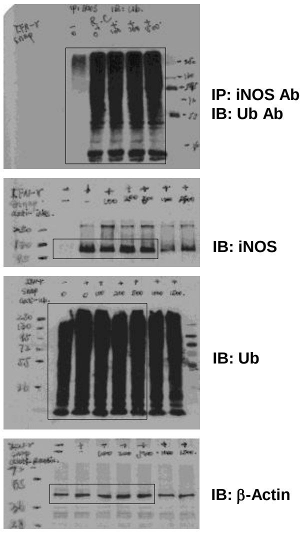

Supplementary Figure 14: full scans of blots in Figure3g and Figure 3h.

Supplementary Figure 15

Fig. 4a

IRF1

IRF4

IRF5

IRF7

IRF8

β-actin

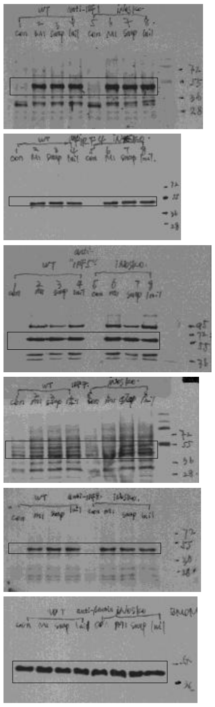

Fig. 4c

IRF5

β-actin

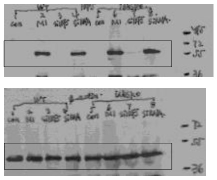

Fig. 4e

Nuc

IRF5

Cyto

IRF5

β-actin

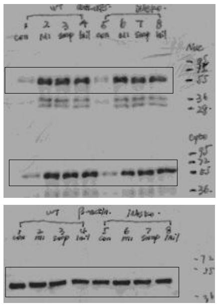

Supplementary Figure 15: full scans of blots in Figure 4a, Figure 4c and Figure 4e.

Supplementary Figure 16

Fig. 5a

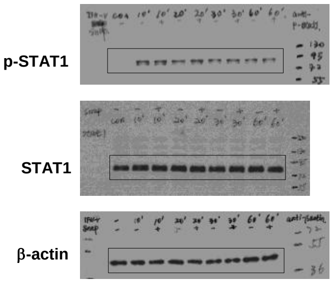

Fig. 5b

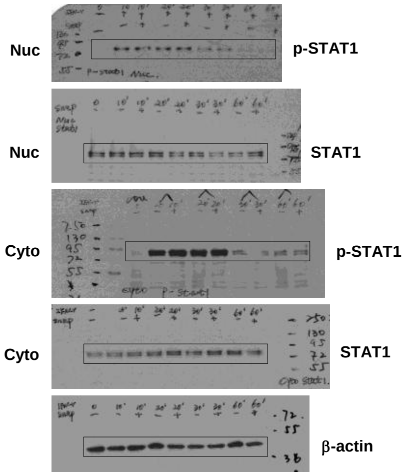

Supplementary Figure 16: full scans of blots in Figure5a and Figure 5b.

Supplementary Figure 17

Fig. 5d

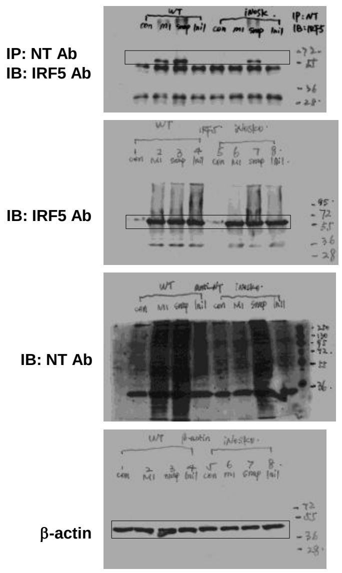

Fig. 5e

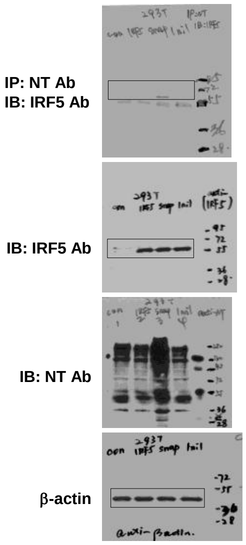

Supplementary Figure 17: full scans of blots in Figure 5d and Figure 5e.

Supplementary Figure 18

Fig. 6b

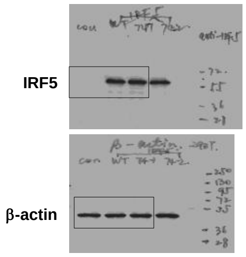

Sup Fig. 8b

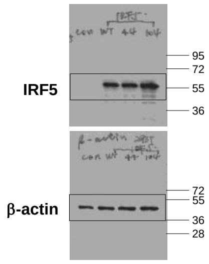

Supplementary Figure 18: full scans of blots in Figure 6b and Supplementary Figure 8b.

Supplementary Table 1. Model variables.

Variable	Description	Range
IL12	IL12 level on log10 scale	0~1
INOS	iNOS level on log10 scale	0~1

Supplementary Table 1. Table 1 defines the two variables of our model.

Supplemetary Table 2. Model Parameter Values.

Parameter	Description	Value
γ_{IL12}	Rate of IL12 reaching its steady state	1
γ_{INOS}	Rate of iNOS reaching its steady state	1
ω_{IL12}	Basal inhibition of IL12	−3.1
$\omega_{IL12,LPS}$	IL12 activation by LPS	1.0230
$\omega_{IL12,INOS}$	IL12 inhibition by iNOS	−1.116
$\omega_{IL12,IL12}$	IL12 self-activation	4.991
ω_{INOS}	Basal activation of iNOS	0.2407
$\omega_{INOS,LPS}$	iNOS activation by LPS	0.2694
$\omega_{INOS,IL12}$	iNOS inhibition by IL12	−1.66
$\omega_{INOS,INOS}$	iNOS self-activation	0
$\omega_{INOS,SNAP}$	iNOS activation by SNAP	0.001
$\theta_{IL12,LPS}$	Basal activation of LPS regulated transcription factor of IL12	1
$\varphi_{IL12,LPS}$	Transcription factor of IL12 activation by LPS	1
$\theta_{IL12,INOS}$	Basal activation of iNOS regulated transcription factor of IL12	−12.5
$\varphi_{IL12,INOS}$	Transcription factor of IL12 activation by iNOS	50
$\theta_{INOS,LPS}$	Basal activation of LPS regulated transcription factor of iNOS	−2.673
$\varphi_{INOS,LPS}$	Transcription factor of iNOS activation by LPS	2.667
$\theta_{INOS,IL12}$	Basal activation of IL12 regulated transcription factor of iNOS	−7.5
$\varphi_{INOS,IL12}$	Transcription factor of iNOS activation by IL12	150
LPS	LPS level (ng mL^{-1}) at log10 scale	-3,-1,0,1,3
SNAP	SNAP level (uM)	0, 500
D_{IL12}	The strength of noise in IL12	0.1250
D_{INOS}	The strength of noise in iNOS	0.0556

Supplementary Table 2. Table 2 lists the parameter values we used for our simulations.
